# Clinical assessment and transcriptome analysis of host immune responses in a vaccination-challenge study using a glycoprotein G deletion mutant vaccine strain of infectious laryngotracheitis virus

**DOI:** 10.3389/fimmu.2024.1458218

**Published:** 2025-01-24

**Authors:** Gayathri Gopakumar, Mauricio J. C. Coppo, Andrés Diaz-Méndez, Carol A. Hartley, Joanne M. Devlin

**Affiliations:** ^1^ Asia-Pacific Centre for Animal Health, Melbourne Veterinary School, Faculty of Science, The University of Melbourne, Parkville, VIC, Australia; ^2^ Escuela de Medicina Veterinaria, Universidad Andrés Bello, Concepción, Chile

**Keywords:** infectious laryngotracheitis virus, vaccination-challenge, RNA-sequencing, transcriptome, immune response, gene enrichment analysis, tracheal mucosae

## Abstract

A glycoprotein-G-deleted live-attenuated vaccine strain of the infectious laryngotracheitis virus (ILTV), ΔgG-ILTV, is safe and efficacious against ILTV challenge. In the current study, the transcriptome of peripheral blood mononuclear cells (PBMCs) of the ΔgG-ILTV-vaccinated group of specific-pathogen-free chickens were compared to those of the nonvaccinated group at 7 days post-vaccination. Tracheal transcriptomes after challenge with virulent ILTV were compared between groups of the non-vaccinated-challenged and the vaccinated-challenged as well as the non-vaccinated-challenged and the uninfected chickens at 4 to 5 days post-challenge. The clinical outcomes after challenge between these groups were also evaluated. Significant differences were observed in the tracheal transcriptome of the non-vaccinated-challenged birds compared to the other two groups. Enriched gene ontologies and pathways that indicated heightened immune responses and impairments to ciliary and neuronal functions, cell junction components, and potential damages to cartilaginous and extracellular components in the trachea of the non-vaccinated-challenged birds were consistent with their severe tracheal pathology compared to the other two groups. On the contrary, the absence of any difference in the tracheal transcriptome between the vaccinated-challenged and the uninfected birds were reflected by the preservation of tracheal mucosal integrity in both groups and mild infiltration of leukocytes in the vaccinated-challenged birds. The results from this study demonstrated that vaccination with ΔgG-ILTV prevented the changes in tracheal transcriptome induced during ILTV challenge, resulting in clinical protection. Additionally, these results also provide insights into the molecular mechanisms underlying the tracheal pathology induced by ILTV infection.

## 1 Introduction

Infectious laryngotracheitis virus (ILTV) is the etiological agent that causes infectious laryngotracheitis (ILT), a respiratory disease affecting poultry (1). Chickens of all age groups are susceptible to ILTV infection. However, outbreaks are commonly reported in birds older than 3 weeks of age.

ILTV infection induces significant pathological changes in the trachea and conjunctiva of birds and causes morbidity, mortality, and reduced egg production in the affected flocks, leading to significant economic losses to poultry industries worldwide (2). Despite the early onset of humoral immune response, disease protection is directly correlated to cell-mediated immune response. Currently, the disease is controlled by the implementation of biosecurity measures and vaccination practices (3).

Several categories of ILT vaccines have been developed over the years (3). These include a glycoprotein G (gG)-deleted live-attenuated vaccine (ΔgG-ILTV) (4) that has been well characterized using *in vitro* and *in vivo* studies (4–11). Initial studies of the ΔgG-ILTV vaccine demonstrated its attenuation (4) and evaluated its levels of immunogenicity and pathogenicity (5). A subsequent study demonstrated the safety and efficacy of this vaccine when delivered through eye-drop and drinking water, which indicated the suitability of the vaccine for mass vaccination of large-scale poultry operations (7). A further study identified the role of ILTV gG as a viral chemokine binding protein (6). The absence of gG during ILTV infection appeared to shift the host immune response away from humoral to the more protective cell-mediated immune response that aligned with the immunomodulatory role of gG (6). The minimum protective dose of the ΔgG-ILTV vaccine for eye-drop delivery has also been determined recently (9). Studies have also assessed the efficacy of drinking water method of administration of the ΔgG-ILTV vaccine in broiler chickens (8) and *in ovo* administration at different doses (10). A more recent study also investigated the latency establishment capacity of the ΔgG-ILTV vaccine (11).

In addition to these clinical investigations, transcriptome-level studies have also been conducted for the ΔgG-ILTV vaccine strain and the parent wild-type strain to evaluate the role of gG during ILTV infection. These studies have demonstrated the influence of gG on the transcription of a selected set of chicken chemokine- and cytokine-encoding genes (12) and the role of gG in the expression of other ILTV genes using qPCR analysis (13). Nevertheless, the genome-wide changes associated with ΔgG-ILTV vaccination in chickens as well as the genes and pathways associated with ΔgG-ILTV vaccine efficacy remain underexplored.

The aim of the current study was to identify the molecular markers associated with ΔgG-ILTV vaccination in peripheral blood mononuclear cells (PBMCs) of vaccinated chickens at 7 days post-vaccination (dpv) and to evaluate the host-response associated with ILTV challenge in the tracheal mucosae of ΔgG-ILTV vaccinated and non-vaccinated SPF chickens at 4 to 5 days post-challenge with a virulent field strain of ILTV.

## 2 Materials and methods

### 2.1 ILTV vaccine and challenge viral strain

Live-attenuated ΔgG-ILTV vaccine (Vaxsafe^®^ ILT, Bioproperties Pty. Ltd.) (4–11) and an Australian virulent strain of ILTV, classified as class 9 ILTV (14, 15), were used in this study. The ΔgG-ILTV vaccine (4) was obtained from the manufacturer and reconstituted as per the manufacturer’s instructions. The class 9 ILTV was first isolated by chorioallantoic membrane inoculation and then propagated on chicken embryo kidney cells and leghorn male hepatoma (LMH) cells (16). Both strains of ILTV were quantitated using plaque-forming unit (PFU) assay on LMH cells prior to inoculation, as described previously (4).

### 2.2 ILTV vaccination and challenge of chickens

The methodology adopted for ILTV vaccination and infection of chickens has been previously described (7). Briefly, 45 white leghorn (SPF) chickens (Australian SPF Services Pty. Ltd.) were wing-tagged after hatching and randomly assigned into three groups of 15. The groups were housed in separate negative pressure isolator units and provided with irradiated feed and water *ad libitum*. At 1 week of age, the birds were vaccinated via eye-drop. Groups 1 and 2 were inoculated with 25 µL of sterile diluent. Group 3 was inoculated with 25 µL of ΔgG-ILTV vaccine (Vaxsafe^®^ ILT) containing a dose of 10^3.8^ plaque-forming units (PFU)/bird.

At 3 weeks post-vaccination, the birds were weighed and inoculated with a virulent field strain of ILTV to assess the level of protection provided by the vaccine. Group 1 was inoculated with sterile diluent and designated as the uninfected negative control group. Groups 2 and 3 were inoculated with the class 9 virulent field strain of ILTV at a dose of 500 PFU/bird and designated as the non-vaccinated-challenged positive control group and the vaccinated-challenged group, respectively. Inoculations were performed via eye-drop (125 PFU/40 µL/each eye) and intratracheal (250 PFU/150 µL) routes, delivering half the dose via each route. After challenge, the birds were monitored daily for clinical signs of ILT or mortality. At 3 and 4 days post-challenge (dpc), the birds were clinically assessed and scored as previously described (5) and as summarized in **Supplementary Table S1**. During clinical assessment, changes in demeanor, dyspnea, and signs of conjunctivitis of the birds were evaluated individually. A score ranging from 0 (normal) to 2 (severely depressed) for demeanor, 0 (normal) to 4 (severe gasping or birds dying due to respiratory distress) for dyspnea, and 0 (normal) to 2 (marked conjunctivitis) for conjunctivitis were used for the assessment.

A total of 10 birds in each group at 4 dpc and five birds in each group at 7 dpc were scheduled for euthanasia for the assessment of protection at the peak of ILTV infection (peak period of ILTV replication and clinical signs) and at a later period during the infection, respectively. Humane killing at the final timepoint or at another timepoint to alleviate suffering was undertaken using intravenous barbiturate (1:1 mixture of pentobarbitone solution and sterile water containing approximately 160 mg of pentobarbitone/mL, resulting in a dose of approximately 160 mg of pentobarbitone/kg body weight) overdose. All birds were weighed after death, prior to post-mortem examination, and percentage change in body weight was calculated based on the difference in weight before challenge and at the time of death. The post-mortem examination included an assessment and scoring of the gross pathology of the tracheal mucosae as described previously (5) and as summarized in **Supplementary Table S2**. The tracheal pathology scores ranged from 0 (absence of pathology) to 4 (severe mucus and hemorrhagic exudates), depending on the severity of the lesions.

All interventions as well as clinical and pathological scoring were performed by trained researchers. To ensure biosecurity, the uninfected group of birds was clinically scored first. Necropsy and sample collection were also conducted first in this group to minimize the risk of cross-contamination. This approach ensured that the researchers remained blinded during the clinical and pathological scoring of both the vaccinated-challenged and non-vaccinated-challenged birds, except for the uninfected group. Additionally, the researchers were blinded during the histopathological assessments of the trachea. Due to ethical considerations and animal welfare concerns, the animal trial in this study was conducted without any repetitions.

### 2.3 Sample collection

At 7 dpv, approximately 0.8 mL of blood was drawn from the basilic vein of both uninfected and vaccinated birds. This blood was collected into separate heparin-coated Vacutainer tubes (BD Biosciences) and stored on ice before isolation of PBMCs. Tracheal samples were collected from birds euthanized at 4 dpc (birds scheduled for euthanasia) or 5 dpc (birds euthanized due to the severity of clinical signs) and from the uninfected birds (4 dpc). A transverse section of the upper trachea (2 to 3 mm thick), immediately distal to the larynx, was collected and fixed in 10% v/v neutral buffered formalin prior to standard histological processing and hematoxylin and eosin staining. Tracheal sections were microscopically examined and scored from 0 (normal) to 5 (severe changes), depending upon the severity of the microscopic lesions as previously described (17) and as summarized in **Supplementary Table S3**. Tracheal mucosal scrapings were collected and stored in 600 µL RLT buffer (RNeasy Mini Kit, Qiagen) containing 1% (v/v) ß-mercaptoethanol and stored at -80°C for nucleic acid extraction.

### 2.4 Isolation of PBMCs

PBMCs were isolated from whole blood samples using Ficoll-Paque PLUS (Cytiva Lifesciences, Marlborough, MA, USA) gradient centrifugation according to the manufacturer’s instructions. Briefly, heparinized blood samples diluted 1:1 in phosphate-buffered solution (PBS) were gently layered over Ficoll-Paque PLUS in 15-mL tubes and centrifuged for 30 min at 400 × *g* at 20°C with no brake. The interface containing PBMCs was collected, washed with phosphate-buffered saline (PBS) twice by centrifugation at 1,000 × *g* for 7 min at room temperature, and the cell pellet was resuspended in 600 µL RLT buffer (RNeasy Mini Kit, Qiagen) containing 1% (v/v) ß-mercaptoethanol prior to storage at -80°C until nucleic acid extraction.

### 2.5 Nucleic acid extraction

Nucleic acids were extracted from the tracheal scrapings and from the PBMCs as mentioned in a previous study (18). For the extraction of nucleic acid from tracheal scrapings, approximately 30 mg of each of the scrapings was homogenized in 600 μL RLT buffer (RNeasy Mini Kit, Qiagen, Hilden, Germany) with 1% (v/v) ß-mercaptoethanol. Then, 200-μL aliquots of the homogenates were subjected to DNA extraction using MagMAX CORE Nucleic Acid Purification Kit (Thermo Fisher Scientific, Waltham, USA) coupled with the KingFisher Flex Purification System (Thermo Fisher Scientific) for automated extractions in 96-well plates as per the manufacturers’ instructions. The eluates (90 μL) were stored at -80°C until qPCR for the detection of ILTV genome copy numbers. The rest of the tracheal homogenates (approximately 300 μL each) and the leukocytes stored in RLT buffer (600 μL each) were subjected to RNA extraction using RNeasy Mini kit (Qiagen). They were then subjected to DNase treatment using Turbo DNA-free kit (Invitrogen, Carlsbad, CA, USA) and concentrated using Zymo RNA Clean and Concentrator-25 (Zymo research Corporation, Irvine, CA, USA) as per the manufacturers’ instructions. The integrity of the RNA eluates (50 μL) was assessed using 4200 Tape Station System (Agilent Technologies, Santa Clara, CA, USA) before storage at -80°C until RNA-seq analysis.

### 2.6 Quantification of ILTV in tracheal scrapings

Quantification of ILTV in tracheal scrapings was performed by employing qPCR analysis of the DNA samples in a Rotor-Gene Q Thermocycler (Qiagen, Hilden Germany). Amplification standard curves were generated using 10-fold serial dilutions of pGEM-T (Promega Corporation, Madison, WI, USA) plasmid carrying the ILTV UL15 gene amplicon (113 bp) (19). The UL15 genome copy numbers (GCN) in the samples were calculated based on cycle threshold values (Ct) using the Rotorgene Q software (version 2.1.0, Qiagen) at a cutoff of 100 genome copies per reaction for the detection and quantification of ILTV DNA.

### 2.7 cDNA library preparation and Illumina sequencing

Total RNA from the PBMCs of four uninfected and eight vaccinated birds and from the tracheal mucosae of eight non-vaccinated-challenged and six each of the uninfected and the most well-protected (based on clinical findings and tracheal pathology) vaccinated-challenged birds, respectively, was subjected to cDNA library preparation and Illumina sequencing at the Australian Genome Research Facility (AGRF, Melbourne, Victoria, Australia). All selected samples had RNA integrity numbers (RIN) of *≥*7. Libraries were prepared using the TrueSeq Stranded mRNA library preparation kit (Illumina Inc., San Diego, CA, USA) and sequenced on NovaSeq (Illumina Inc.) using the Illumina DRAGEN BCL Convert 07.021.645.4.0.3 pipeline to generate 150-bp paired-end reads.

### 2.8 RNA-seq data pre-processing and differential gene expression analysis

Analyses of RNA-seq data were performed using the web-based bioinformatics platform GALAXY at *usegalxy.au* following the published *Galaxy workflow* (20–22). The quality of the raw data sets was analyzed using FastQC (Galaxy Version 0.74+galaxy0). Adaptor sequences were removed using Trimmomatic (Galaxy Version 0.36.6) and read pairs with PHRED quality score >20 and length >20 bp were retained. Using the Ensemble release 106 of the annotated chicken (*Gallus gallus*) genome (FASTA and gene transfer format) as reference, mapping of the reads was performed using RNA STAR (Galaxy Version 2.7.10b+galaxy3). Exon-level read counts of the BAM outputs were performed using featureCounts (Galaxy Version 2.0.3+galaxy1) and paired-end reads that mapped to multiple locations or reads with a minimum mapping quality score (minMOS) <10 were eliminated. Differential gene expression (DGE) analysis was performed using Deseq2 (Galaxy Version 2.11.407 +galaxy2) using the default GALAXY settings. Protein-coding genes of the chicken, differentially expressed in pair-wise comparisons between the different groups at *P*-adj value (*P*-value corrected using Benjamini–Hochberg procedure for the Wald statistic) <0.01 and with greater than or equal to twofold change (log_2_FC1) in expression, were considered significant. To assess the stability of the RNA-seq assay, the transcription of the 10 constitutively transcribed house-keeping genes reported for chicken trachea (23) was examined in the pair-wise comparisons between the different groups.

### 2.9 Gene ontology analysis

Gene ontology (GO) analysis was conducted using PANTHER (version 17.0) (24, 25). GO terms enriched for biological processes (BP), molecular functions (MF), and cellular components (CC) separately with the upregulated or downregulated genes in the trachea of the non-vaccinated-challenged birds were identified and compared to those of the vaccinated-challenged and the uninfected birds (18). The most enriched GO terms selected based on a false discovery rate (FDR) <0.05 and with fold enrichment values >1 were summarized using REVIGO (26) for the removal of redundant GO terms and reported.

### 2.10 Pathway and protein class analysis

Pathway and protein class analysis were performed using PANTHER (version 17.0) (24, 25). Reactome pathways and PANTHER protein classes enriched separately with the upregulated and downregulated genes in the trachea of the non-vaccinated-challenged birds compared to those of the other two groups at FDR <0.05 were identified and reported.

### 2.11 Statistical analysis and data visualization

Mann–Whitney *U*-test was used for the comparisons of clinical scores, tracheal gross pathology scores, and tracheal histopathology scores. Fisher’s exact test was used for the comparison of proportions between groups. Normality of the data for weigh change and ILTV genome copy numbers (GNCs) were assessed using Shapiro–Wilk’s test. One-way ANOVA corrected for multiple comparisons using Tukey’s test was used for the comparison of the normally distributed percentage weight change data. Kruskal–Wallis test corrected for multiple comparisons using Dunn’s test was used for the comparison of the non-normally distributed ILTV GCN data. Statistical analyses were performed using Prism-GraphPad version 9.4.1. Data visualization was performed using R version 4.0 and SRplot.

## 3 Results

### 3.1 Vaccinated birds were protected from ILTV challenge

**Figure 1:**
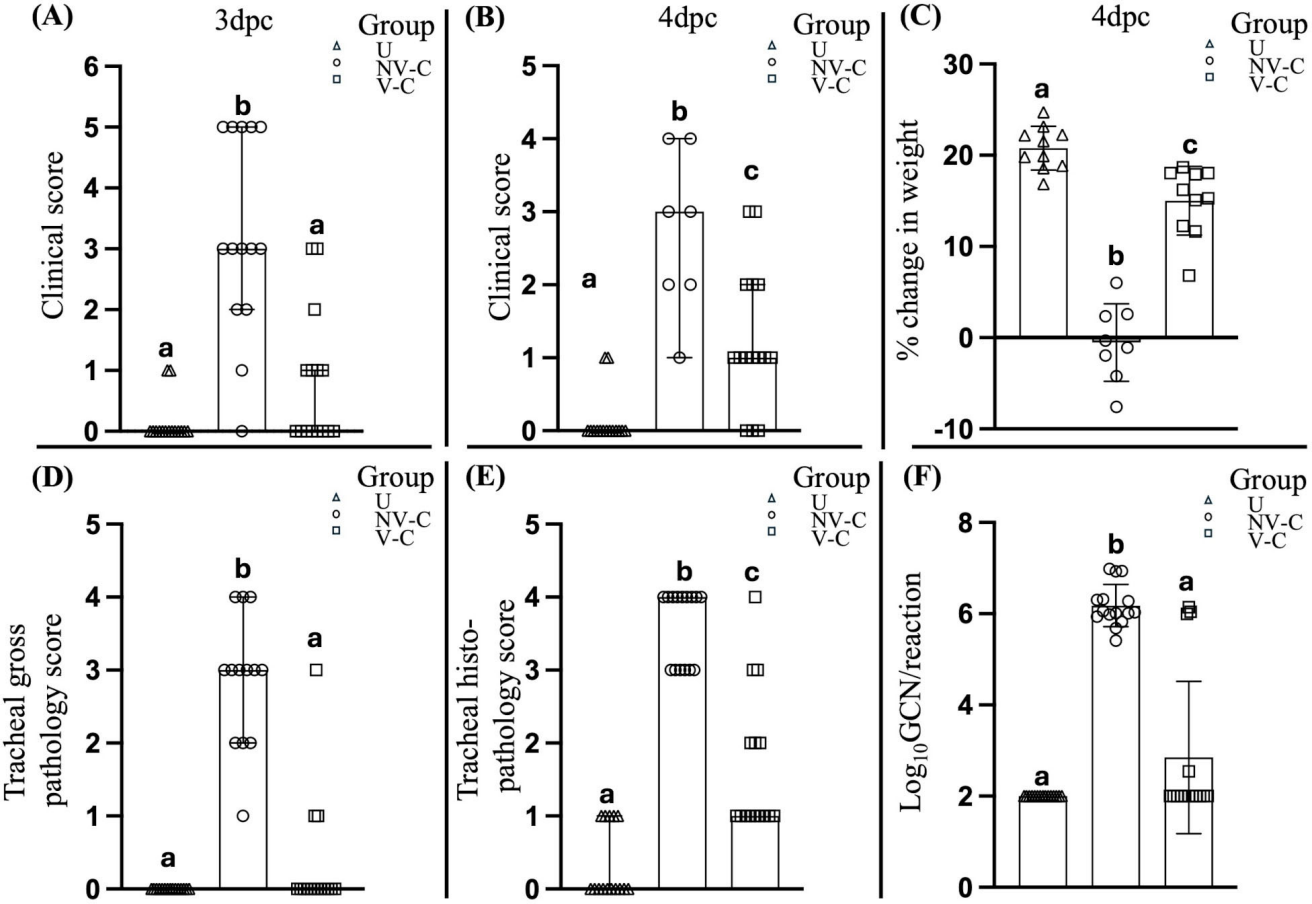
Clinical assessment of the uninfected (negative control) birds, non-vaccinated-challenged birds, and vaccinated-challenged birds after challenge with virulent ILTV. Scatter plot with bars **(A, B)** represents the clinical scores of the birds at 3 and 4 days post-challenge (dpc) respectively. Scatter plot with bars indicating **(C)** percentage change in body weight, **(D)** tracheal gross pathology scores, **(E)** tracheal histopathology scores, and **(G)** ILTV genome copy number (GCN)/reaction; lines at median with 95% confidence interval in **(A, B, D–F)** and line at mean with standard deviation in **(C)**. Log_10_ GCN/reaction values of the samples that tested negative for ILTV DNA **(F)** were adjusted to 2, as 100 copies per reaction were used as the cutoff for the detection and quantification of ILTV DNA; values marked with the same superscript letter above each group in each panel were not significantly different (*P* > 0.05); U, uninfected; NV-C, non-vaccinated-challenged; V-C, vaccinated-challenged; dpc, days post-challenge.

The vaccinated birds had significantly (*P* < 0.05) less severe clinical signs than the non-vaccinated-challenged birds at 3 (*P* = 0.0002) and 4 dpc (*P* = 0.0087) (**Figures 1A, B**). Significant differences were also observed in the cumulative mortality between the vaccinated-challenged and the non-vaccinated-challenged birds (*P* = 0.002). While there were no mortalities among the vaccinated-challenged birds (0% cumulative mortality at 7 days post-challenge), the non-vaccinated-challenged group experienced two sudden deaths—one bird at 3 dpc and another at 4 dpc. Additionally, six birds in the non-vaccinated-challenged group were subjected to humane killing at 3 dpc due to severe clinical signs, resulting in a cumulative mortality rate of 53.33% by 7 days post-challenge, in accordance with the specified interventions outlined in the animal ethics approval. These birds were assigned a summed clinical score of 5, as individual scoring was not feasible due to the immediate need for euthanasia based on ethical considerations.

The vaccinated-challenged birds also had a significantly greater weight gain than the non-vaccinated-challenged birds (*P* < 0.0001) at 4 dpc (**Figure 1C**).

### 3.2 Vaccinated birds had reduced tracheal pathology and ILTV GCN after challenge with virulent ILTV

**Figure 2:**
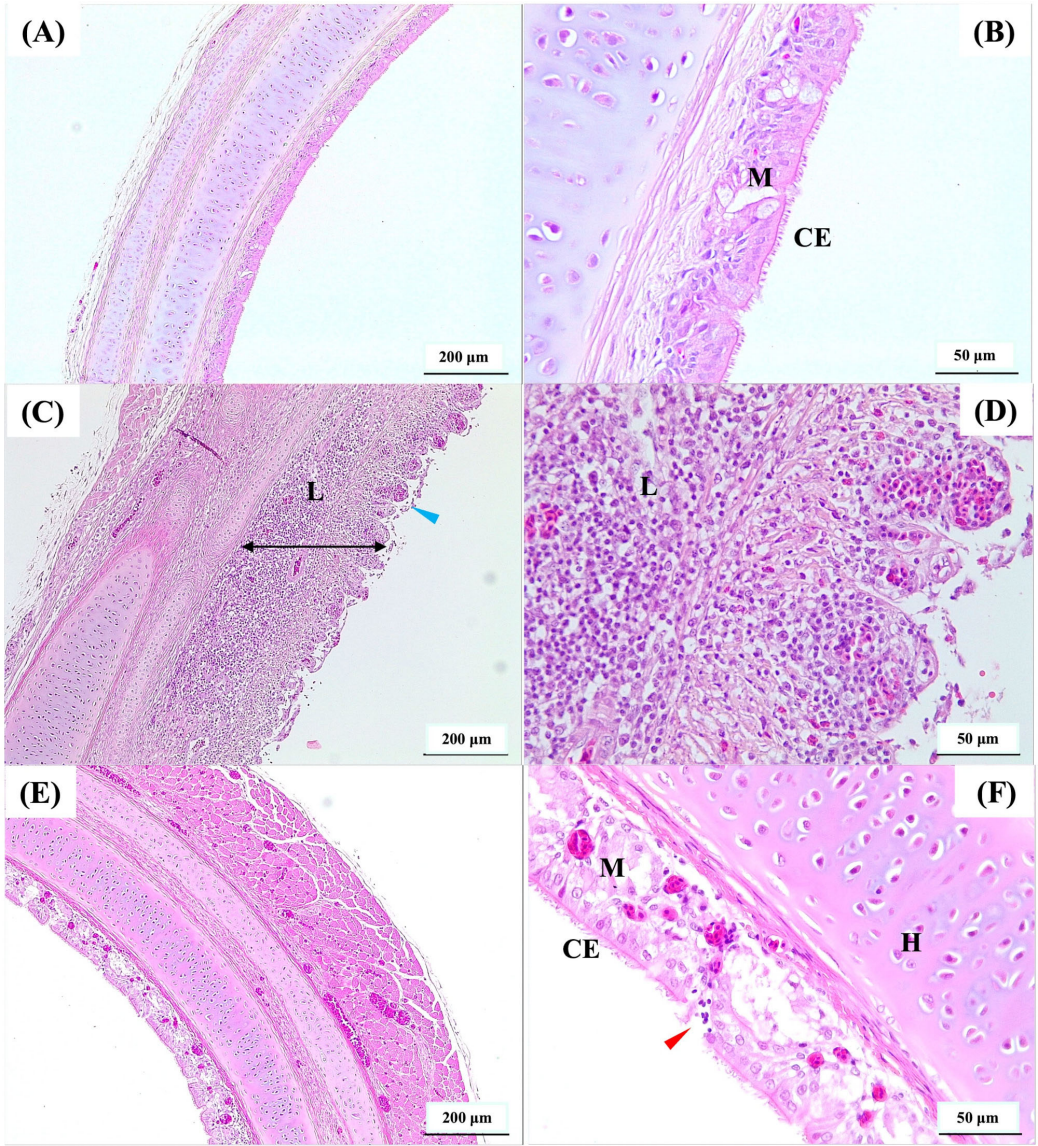
Photomicrographs of hematoxylin and eosin-stained cross-sections of the upper trachea. **(A, B)** represent uninfected, **(C, D)** represent non-vaccinated-challenged, and **(E, F)** represent vaccinated-challenged birds with median histopathology scores of 0, 4, and 1, respectively. The presence of abnormal epithelium without cilia (blue arrowhead) and increased thickness of mucosa (double headed black arrow) due to edema and heavy infiltration of leukocytes (L) was observed in **(C, D)**. Ciliated pseudostratified columnar epithelial cells (CE), mucus or goblet cells (M), and mild infiltration of leukocytes (red arrowhead) were observed in **(E, F)**. Scale bars in **(A, C, E)** correspond to 200 μm and in **(B, D, F)** indicate 50 μm. H, hyaline cartilage.

The vaccinated birds had significantly lower (*P* < 0.05) tracheal gross pathology and histopathology scores after challenge compared to the non-vaccinated-challenged birds (**Figures 1D, E**). The non-vaccinated-challenged birds had a median gross tracheal pathology score of 3, corresponding to a large amount of bloody mucus with the occasional presence of diphtheritic plaques, while the vaccinated-challenged birds had a score of 0 (**Figure 1D**). A median tracheal histopathology score of 4 in the non-vaccinated-challenged birds (**Figure 1E**) reflected severe changes to the trachea. Compared to the uninfected birds (**Figures 2A, B**), the tracheae of the non-vaccinated-challenged birds exhibited heavy infiltration of leukocytes accompanied by edema or cellular exudate and an absence of normal epithelium (**Figures 2C, D**). On the other hand, the median tracheal histopathology score of 1 in the vaccinated challenged birds (**Figure 1E**) reflected minimal changes, with intact ciliated columnar epithelial cells and mild leukocyte infiltration (**Figures 2E, F**).

Significant differences (*P* < 0.05) between the vaccinated-challenged and the non-vaccinated-challenged birds were also observed in the proportion of birds that tested positive for ILTV in the trachea (**Figure 1F**). ILTV was only detected in 4/15 of vaccinated birds but detected in each of the 15 non-vaccinated-challenged birds. Additionally, the genomic copy numbers (GCNs) of ILTV were significantly lower (*P* = 0.0002) in the vaccinated birds compared to the non-vaccinated-challenged birds (**Figure 1F**).

### 3.3 Differential gene expression analysis

RNA-seq reads after quality assessment and trimming were mapped to the reference chicken genome (**Supplementary Tables S4**, **S5**). The global gene expression pattern of the PBMCs, visualized via principal component analysis (PCA), showed no clear distinction between the vaccinated and the non-vaccinated (uninfected) groups (**Supplementary Figure S1**). No difference was observed in the gene expression of the PBMCs of the vaccinated birds at 7 dpv compared to the uninfected group, at *P*-adj value <0.01 and greater than or equal to twofold change (log_2_FC 1) in expression.

**Figure 3:**
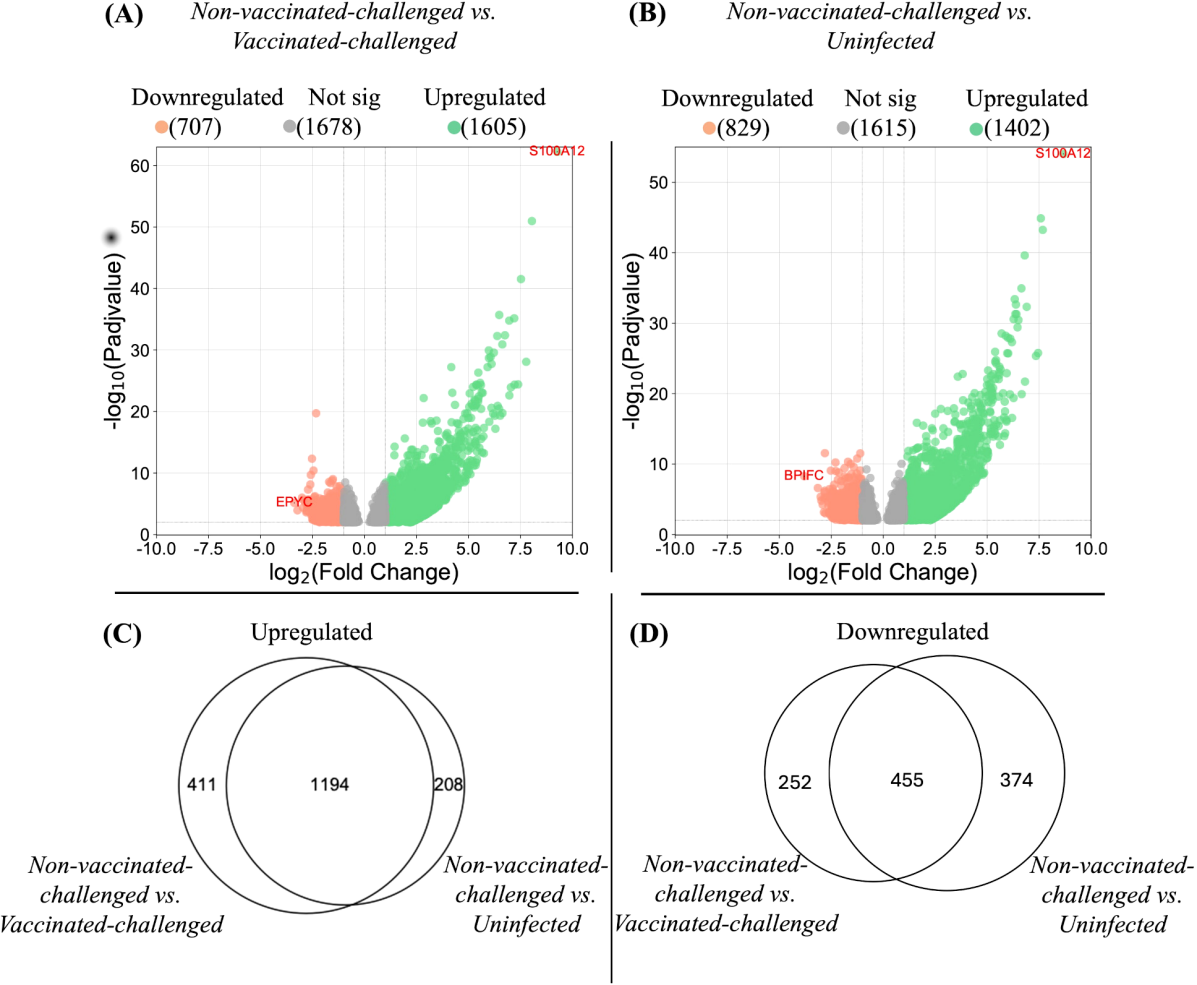
Differentially expressed genes in tracheal mucosae. **(A, B)** represent the differentially expressed genes in the tracheal mucosae of the non-vaccinated-challenged group compared to the vaccinated-challenged and to the uninfected groups, respectively. Genes differentially regulated at *P*-adj value <0.01 and log_2_ fold change ≥1 were considered significantly upregulated, and genes differentially regulated at *P*-adj value <0.01 and log_2_ fold change ≤-1 were considered significantly downregulated. The position of the most significantly up- and downregulated gene in each comparison is labeled with gene symbols. Proportional Venn diagrams **(C, D)** represent the number of differentially expressed genes in the tracheal mucosae of the non-vaccinated-challenged group compared to the vaccinated-challenged and/or the uninfected groups. **(C)** Upregulated genes. **(D)** Downregulated genes. The numbers in the intersection of the circles represent the number of genes up- or downregulated in the non-vaccinated-challenged compared to the vaccinated-challenged or the uninfected group, while the numbers outside the intersection of the circles represent the uniquely up- or downregulated genes in the non-vaccinated-challenged group compared to each of the groups.

The global gene expression pattern of the tracheal mucosae of the vaccinated-challenged and the uninfected groups were similar at 4 or 5 dpc (**Supplementary Figure S2**). No genes exhibited differential regulation between the groups at *P*-adj value < 0.01 and a fold change in expression of at least twofold (log_2_FC). On the other hand, the non-vaccinated-challenged group segregated into a distinct cluster, separated from the other two groups. A greater number of genes was upregulated than downregulated in the non-vaccinated-challenged group compared to the other two group (**Figures 3A, B**, respectively), and a large proportion of the differentially expressed genes observed in the non-vaccinated-challenged group was shared between the vaccinated-challenged and the uninfected groups (**Figures 3C, D**, respectively). Of the 10 most stable and reliable chicken tracheal housekeeping genes (23), the majority were found to be constitutively transcribed, with no significant difference in their expression in the pairwise comparisons between the non-vaccinated-challenged and the uninfected birds (8/10 genes) and the non-vaccinated-challenged and the vaccinated-challenged bids (7/10), thus validating the stability of the RNA-seq results (**Supplementary Tables S6**, **S7**).

### 3.4 Genes most significantly upregulated in the trachea of the non-vaccinated-challenged birds were mostly related to immune response, cell proliferation, and cell activation

The gene encoding *S100A12*, a chemoattractant protein expressed in granulocytes, exhibited the highest level of upregulation in the tracheal mucosa of the non-vaccinated-challenged birds compared to the vaccinated-challenged (log_2_FC 9.27) and uninfected birds (log_2_FC 8.67). This inflammatory marker protein is involved in chemotaxis, production of cytokines, cell proliferation and differentiation, and induction of oxidative stress (27, 28). Several other genes involved in immune and inflammatory responses were also upregulated in the non-vaccinated-challenged birds and constituted the largest category of genes by function, among the top 20 upregulated genes (**Table 1**). This included the genes encoding several chemokines and cytokines and/or their receptors, immunoglobulin receptors, *TLR15*, *ACOD*, a mitochondrial enzyme that regulates immunometabolism, inflammation, and infection in several species (29), and the GTPase, *RAB3C*, involved in the recycling of phagocytosed MHC class I complex (in humans) (30). The other categories of genes in the list included those that encode proteins involved in the proliferation and activation of cells (granulocytes, macrophages, osteoclasts, and chondrocytes), regulation of apoptosis and necroptosis, extracellular matrix (ECM) remodeling (metallopeptidases), and vascular repair (*NOXO1*) and fluid homeostasis (*SCNNID*).

**Table 1:** Top 20 upregulated genes in trachea of the non-vaccinated-challenged birds compared to the vaccinated-challenged or uninfected birds.

Gene name	Non-vaccinated-challenged vs.			
	Vaccinated-challenged		Uninfected	
	*P*-adj	FC^*^	*P*-adj	FC^*^
Immune and inflammatory response				
S100 calcium binding protein A12	5.90E-63	9.27	9.41E-55	8.67
Aconitate decarboxylase 1	8.23E-29	7.78	1.71E-26	7.45
Fc fragment of IgE receptor II	2.89E-42	7.53	5.72E-44	7.68
Chemokine (C–C motif) ligand 4	3.89E-25	7.39	1.20E-20	6.66
Interleukin 8-like 1	1.18E-24	7.04	N	N
C–X–C motif chemokine receptor 1	1.21E-31	6.63	3.92E-30	6.46
Interleukin 1, beta	1.22E-21	6.40	N	N
C–C motif chemokine ligand 17	5.22E-33	6.39	1.15E-35	6.65
Cytokine receptor common subunit beta-like	N	N	2.28E-33	6.39
Lymphocyte antigen 96	N	N	2.70E-31	6.28
Immunoglobulin superfamily member 1	N	N	3.92E-34	6.33
Toll-like receptor 15	4.89E-20	6.27	4.29E-20	6.27
RAB3C, member RAS oncogene family	6.35E-18	6.30	2.86E-17	6.20
Proliferation, differentiation, or activation of cells				
Colony stimulating factor 3 receptor	1.07E-51	8.05	1.30E-45	7.58
MAS-related GPR, member H	4.26E-25	7.22	4.49E-26	7.35
Avidin	2.39E-23	6.97	1.99E-22	6.82
Dendrocyte expressed seven transmembrane protein	3.41E-21	6.47	N	N
Colony stimulating factor 2 receptor b common subunit	N	N	5.09E-32	6.41
Colony stimulating factor 2 receptor a subunit	N	N	5.09E-32	6.36
Regulation of apoptosis or necroptosis				
Mixed lineage kinase domain like pseudokinase	6.85E-36	7.20	4.81E-33	6.91
Serpin peptidase inhibitor, clade B, member 10 B	4.74E-20	6.52	N	N
Leukocyte ribonuclease A-2	1.98E-36	6.48	2.42E-40	6.81
ECM remodeling and tissue repair				
ADAM metallopeptidase domain 8	1.63E-35	6.96	3.55E-31	6.51
Matrix metallopeptidase 9	4.01E-33	6.76	N	N
NADPH oxidase organizer 1	N	N	5.02E-28	6.19
Fluid homeostasis				
Sodium channel epithelial 1 delta subunit	1.77E-20	6.64	N	N

* Log_2_ fold change; *P*-adj-value <0.01 and log_2_ fold change ≥1 was considered significant.

*N*, not in the top 20 list.

### 3.5 Genes most significantly downregulated in the trachea of the non-vaccinated-challenged birds were mostly related to extracellular matrix organization and cell adhesion

Genes encoding *EPYC* and *BPIFC* were the most downregulated genes in the trachea of the non-vaccinated-challenged birds compared to the vaccinated-challenged (log_2_FC = -3.37) and the uninfected birds (log_2_FC = -3.80), respectively. The former is a proteoglycan that interacts with collagen fibers and ECM proteins and aids in cartilage matrix organization (31), while the latter is a palate, lung, and nasal epithelium clone (*PLUNC*) protein expressed by airway epithelial and/or mucus cells, with innate immune functions (32, 33). Of the top 20 downregulated genes in non-vaccinated-challenged birds, genes that encode proteins involved in tissue remodeling and ECM organization constituted the largest category of genes by function (**Table 2**). This included the genes encoding *ACAN*, a proteoglycan involved in cartilage remodeling and ECM organization (34), the bone matrix non-collagenous protein *OC3* (35), the cartilage matrix protein *CHAD* (36), growth factors such as *CTGFL* and *GDF10* and the cell-line-derived transforming sequencing *MCF2* with roles in bone morphogenesis and remodeling (37–39), *AVPR1A* involved in hematopoiesis (40), and the muscle protein *MYH1C* (41). The second major category of genes included those that encode proteins with roles in cell adhesion. Genes related to other functions in the list included those with neuronal functions, transmembrane transport, immune and inflammatory responses, steroid metabolism, protein production, and protein-to-protein interactions.

**Table 2:** Top 20 downregulated genes in the trachea of the non-vaccinated-challenged birds compared to the vaccinated-challenged or uninfected birds.

Gene name (gene symbol)	Non-vaccinated-challenged vs.			
	Vaccinated-challenged		Uninfected	
	*P*-adj	FC^*^	*P*-adj	FC^*^
Connective tissue/muscle remodeling and extracellular matrix organization				
Epiphycan	7.25E-06	-3.37	8.90E-04	-2.59
Aggrecan	1.22E-04	-2.77	N	N
Osteocalcin-like protein OC3	4.08E-06	-2.63	6.24E-07	-2.81
Connective tissue growth factor-like	7.30E-09	-2.60	N	N
Arginine vasopressin receptor 1A	N	N	5.78E-06	-2.60
Chondroadherin	N	N	1.12E-03	-2.60
Growth differentiation factor 10	6.11E-05	-2.54	N	N
MCF.2 cell line derived transforming sequence	4.63E-13	-2.52	N	N
Myosin, heavy chain 1C, skeletal muscle	4.63E-13	-2.51	N	N
Cell adhesion				
Ankyrin repeat domain 34C	N	N	1.58E-05	-2.98
Fibronectin type III domain containing 1	5.34E-06	-2.87	6.36E-06	-2.84
Cellular communication network factor 3	2.40E-04	-2.80	6.51E-04	-2.62
DS cell adhesion molecule	4.88E-08	-2.72	N	N
EGF like repeats and discoidin domains 3	N	N	1.96E-05	-2.65
Integrin subunit beta like 1	N	N	8.27E-05	-2.63
Zona pellucida sperm-binding protein 3	4.17E-05	-2.75	N	N
Tubulointerstitial nephritis antigen	6.71E-04	-2.56	N	N
Neuronal functions				
Adhesion G protein-coupled receptor B1	3.36E-05	-2.73	N	N
Proteolipid protein 1	5.56E-04	-2.53	N	N
CUB and Sushi multiple domains 3	N	N	7.01E-04	-2.85
Gap junction protein gamma 2	N	N	9.97E-07	-2.82
Pleiotrophin	N	N	3.96E-06	-2.66
Transmembrane transport				
Solute carrier family 5 member 8	1.90E-06	-2.71	9.19E-06	-2.53
Solute carrier family 38 member 4	2.01E-10	-2.58	2.88E-12	-2.80
Immune and inflammatory response				
C1q and tumor necrosis factor related protein 8	1.02E-06	-3.00	2.53E-07	-3.14
Interleukin 17 receptor E like	2.33E-06	-2.94		
BPI fold containing family C, member B	N	N	5.53E-09	-3.80
Bradykinin receptor B1	N	N	3.60E-07	-2.58
Steroid metabolism				
Hydroxy-delta-5-steroid dehydrogenase, 3 beta- and steroid delta-isomerase 1	N	N	7.23E-06	-2.79
Protein production and protein-to-protein interactions				
Coiled-coil domain containing 129	N	N	4.58E-06	-3.00
Chromosome 10 open reading frame	4.35E-05	-2.96	5.89E-05	-2.91
Chromosome 2 open reading frame	1.51E-04	-2.79	N	N

* Log_2_ fold change; *P*-adj-value <0.01 and log_2_ fold change ≤-1 was considered significant.

*N*, not in the top 20 list.

### 3.6 Genes differentially regulated in the non-vaccinated-challenged birds included many with immune-related functions

A large number of genes with role in immune response were upregulated in the tracheae of the non-vaccinated-challenged birds compared to the birds in the other two groups. Of these, the largest category was constituted by chemokines, cytokines, and their receptors (**Supplementary Table S8**). The chemokine-encoding genes most significantly upregulated in the non-vaccinated-challenged group compared to the vaccinated-challenged and the uninfected groups were *IL8L1* (log_2_FC = 7.04) and *CCL17* (log_2_FC = 6.65), respectively. While the expression of genes encoding several other interleukins, interleukin receptors, -CC and -CXC families of chemokines, and chemokine receptors, were upregulated in the non-vaccinated-challenged group compared to either of the other groups, the gene for *XCL1* (log_2_FC = 1.90), one of the two members of the C-family of chemokines and *CX3CR1* (log_2_FC = 2.21), the receptor for the only known CX3C subfamily of chemokines, was upregulated uniquely compared to the uninfected group (42, 43). Genes encoding several toll-like receptor family members (*TLR15*, *TLR4*, *TLR7*, *TLR2B*, *TLR1B*, and *TLR2A)* and MHC-related proteins, including MHC class I, MHC class II, and MHC B-G antigen-related proteins, were also upregulated in the non-vaccinated-challenged group. Upregulated interferon-encoding genes included *IFNκL1* and *IFN-γ*, which were upregulated compared to both the vaccinated-challenged (log_2_FC = 5.08 and 4.51, respectively) and the uninfected groups (log_2_FC = 4.97 and 4.14, respectively), while the gene for *IFN-ω1* was upregulated uniquely when compared to the former (log_2_FC = 2.30). Genes encoding several interferon regulatory factors and interferon-induced proteins constituted the other interferon-related genes that were upregulated in the non-vaccinated-challenged group. Among the genes encoding nuclear factors that were upregulated in the non-vaccinated-challenged birds, genes for *NFE2*, which regulate antioxidant response elements during oxidative airway disorders (44, 45), were upregulated when compared to both the vaccinated-challenged and the uninfected groups (log_2_FC 1.80 and 1.53, respectively). Additionally, genes encoding *NFIL3* and *NFκB2* (log_2_FC = 1.37 and 1.18, respectively) were also upregulated in the non-vaccinated-challenged birds compared to the vaccinated-challenged birds. The other upregulated immune-related genes included those encoding several TNF superfamily members, TNF-alpha-induced proteins, and CD molecules as well as complement-related proteins.

The largest category of genes with immune-related functions that was downregulated in the non-vaccinated-challenged group was that of “complement components”, which included complement factors and C1q tumor necrosis factor (TNF)-related superfamily of proteins (CTRP) (**Supplementary Table S9**). Downregulated chemokines and/or chemokine-receptor-encoding genes included *CCL20* (log_2_FC = -1.86, -2.52) and receptors for interleukin 1 (*IL1R1*, log_2_FC = -1.07, -1.08) and 17 (*IL17REL*, log_2_FC = -2.94, -2.13) and an accessory protein for interleukin 1 receptor (*IL1RAPL2*, log_2_FC = -2.10, -1.74). In addition to this, a few immunoglobin-related genes and CD molecules were also downregulated.

### 3.7 Gene ontology terms related to immune response, cytokine and chemokine activity, and membrane were the most upregulated terms in the non-vaccinated-challenged birds

**Figure 4:**
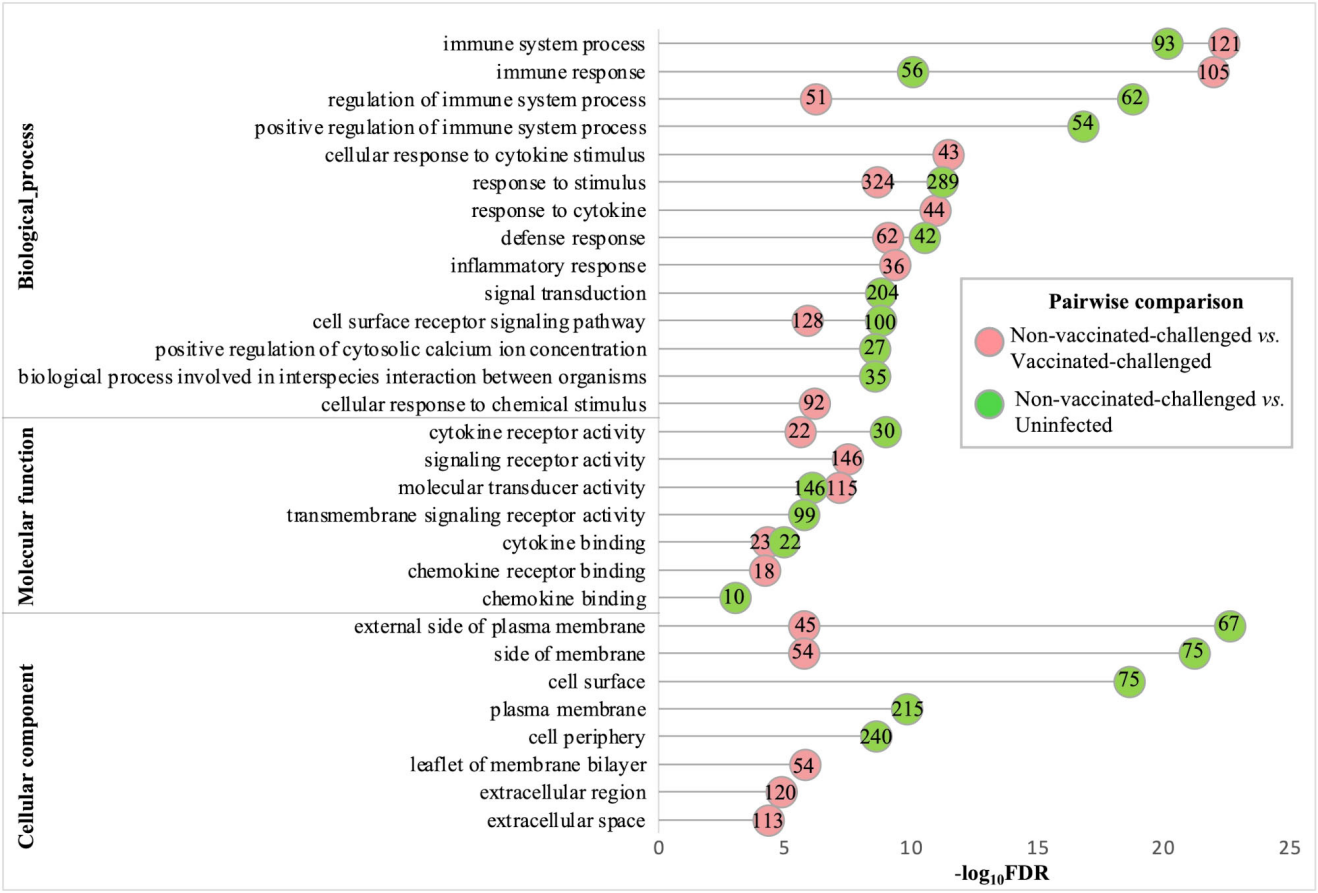
Gene ontologies most significantly enriched (FDR <0.05 and fold enrichment >1) with the upregulated genes of the non-vaccinated-challenged group compared to the vaccinated-challenged and the uninfected groups. The top 10 (FDR < 0.05) biological process (BP), five molecular functions (MF), and five cellular components (CC) terms are listed. The numbers inside the circles denote the number of genes enriched for the GO terms.

The gene ontology analysis of the upregulated genes of the non-vaccinated-challenged group compared to the vaccinated-challenged and to the uninfected groups revealed comparable profiles for the most enriched (FDR <0.05 and fold enrichment >1) BP, MF, and CC root categories. A total of 76, 18, and 10 GO terms for BPs, MFs, and CCs, respectively, were enriched with the genes upregulated in the non-vaccinated-challenged birds compared to the vaccinated-challenged birds, while 59, eight, and nine GO terms for the same, respectively, were enriched with the genes upregulated in the non-vaccinated-challenged birds compared to the uninfected birds. The largest compartments in the REVIGO summarized tree maps for the upregulated GOs were constituted by the representative BP terms “immune response” and “regulation of immune response”, MF terms for “cytokine binding” and “cytokine receptor activity”, and the CC term for “external side of plasma membrane” and “side of membrane” (**Supplementary Figures S3**-**S8**). The GO terms enriched most significantly with the upregulated genes in the non-vaccinated-challenged group compared to the vaccinated-challenged and the uninfected groups are shown in **Figure 4**. The top 10 upregulated BPs were mostly related to immune, defense, and inflammatory responses, signaling, and response to cytokines. The top five MFs were mostly related to cytokine and chemokine activity and binding, while the top five CCs mostly highlighted extracellular components and membranes (**Figure 4**). The upregulated BP, MF, and CC terms in the non-vaccinated-challenged group with the greatest fold enrichment (FE) compared to those of the vaccinated challenged birds were the terms for “cellular response to biotic stimulus” (16/21 genes, FE = 8.6), “complement binding” (5/5 genes, FE = 11.28), and “intermediate filament” (10/34 genes, FE = 3.32), respectively, while those compared to the uninfected birds were the terms for “positive regulation of adaptive immune response” (4/5 genes, FE = 10.33), “MHC protein binding” (5/6 genes, FE = 10.76), and “external side of plasma membrane” (67/146 genes, FE = 5.93), respectively.

### 3.8 Gene ontology terms related to developmental processes, extracellular structure organization, channel activity, and adhesion were the most downregulated terms in the non-vaccinated-challenged birds

**Figure 5:**
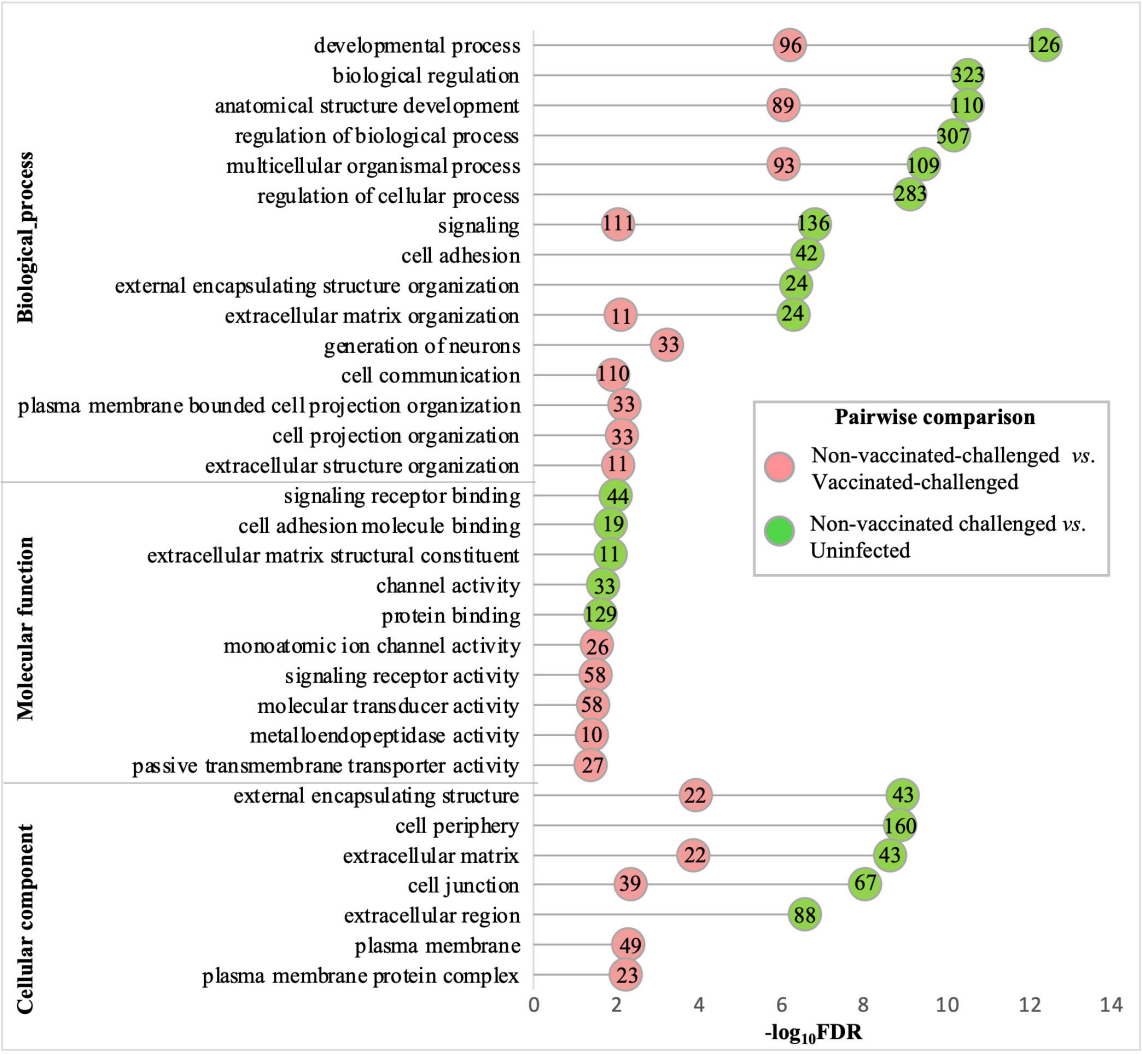
Gene ontologies most significantly enriched (FDR <0.05 and fold enrichment >1) with the downregulated genes of the non-vaccinated-challenged group compared to the vaccinated-challenged and the uninfected groups. The top 10 (FDR < 0.05) biological process (BP), five molecular functions (MF), and five cellular components (CC) terms are listed. The numbers inside the circles denote the number of genes enriched for the GO terms.

The gene ontologies enriched with the downregulated genes in the non-vaccinated-challenged group compared to the other two groups were similar for the most enriched (FDR <0.05 and fold enrichment >1) BP, MF, and CC categories. A total of 23, six, and 13 GO terms for BPs, MFs, and CCs, respectively, were enriched with the genes downregulated in the non-vaccinated-challenged birds compared to the vaccinated-challenged birds, while 40, 12, and nine GO terms for the same, respectively, were enriched with the genes downregulated in the non-vaccinated-challenged birds compared to the uninfected birds. The largest compartments in the REVIGO summarized tree maps for the GOs downregulated in non-vaccinated-challenged group compared to the vaccinated-challenged and the uninfected groups, respectively, were constituted by the representative BP terms “anatomical structure development” and “regulation of multicellular organismal development”, MF terms “voltage-gated monoatomic ion channel activity” and “cell adhesion molecule binding”, and the CC term “extracellular matrix” (**Supplementary Figures S9**-**S14**). The GO terms enriched most significantly (FDR < 0.05) with the downregulated genes in the non-vaccinated-challenged group compared to the vaccinated-challenged and the uninfected groups are shown in **Figure 5**. The top 10 downregulated BPs were mostly related to various developmental processes, extracellular structure organization, cell adhesion, cell communication, signaling, and generation of neurons. The top five MFs were mostly related to channel or transporter activity and protein binding, including receptors and cell adhesion molecules, while the top five CCs mostly highlighted extracellular components and membranes including cell junctions (**Figure 5**). The downregulated BP, MF, and CC terms in the non-vaccinated-challenged group with the greatest fold enrichment (FE) compared to the vaccinated challenged birds were the terms for “extracellular matrix organization” (11/62 genes, FE = 4.54), “metalloendopeptidase activity” (10/69 genes, FE = 3.71), and “post-synaptic density” (9/57 genes, FE = 4.04), respectively, while those compared to the uninfected birds were the terms for “cognition” (4/9 genes, FE = 9.71), “heparin binding” (6/21 genes, FE = 6.24), and “external encapsulating structure” (43/254 genes, FE = 3.7).

### 3.9 Pathways mainly involved in immune response were upregulated, while ECM and collagen organization were downregulated in the non-vaccinated-challenged birds

**Figure 6:**
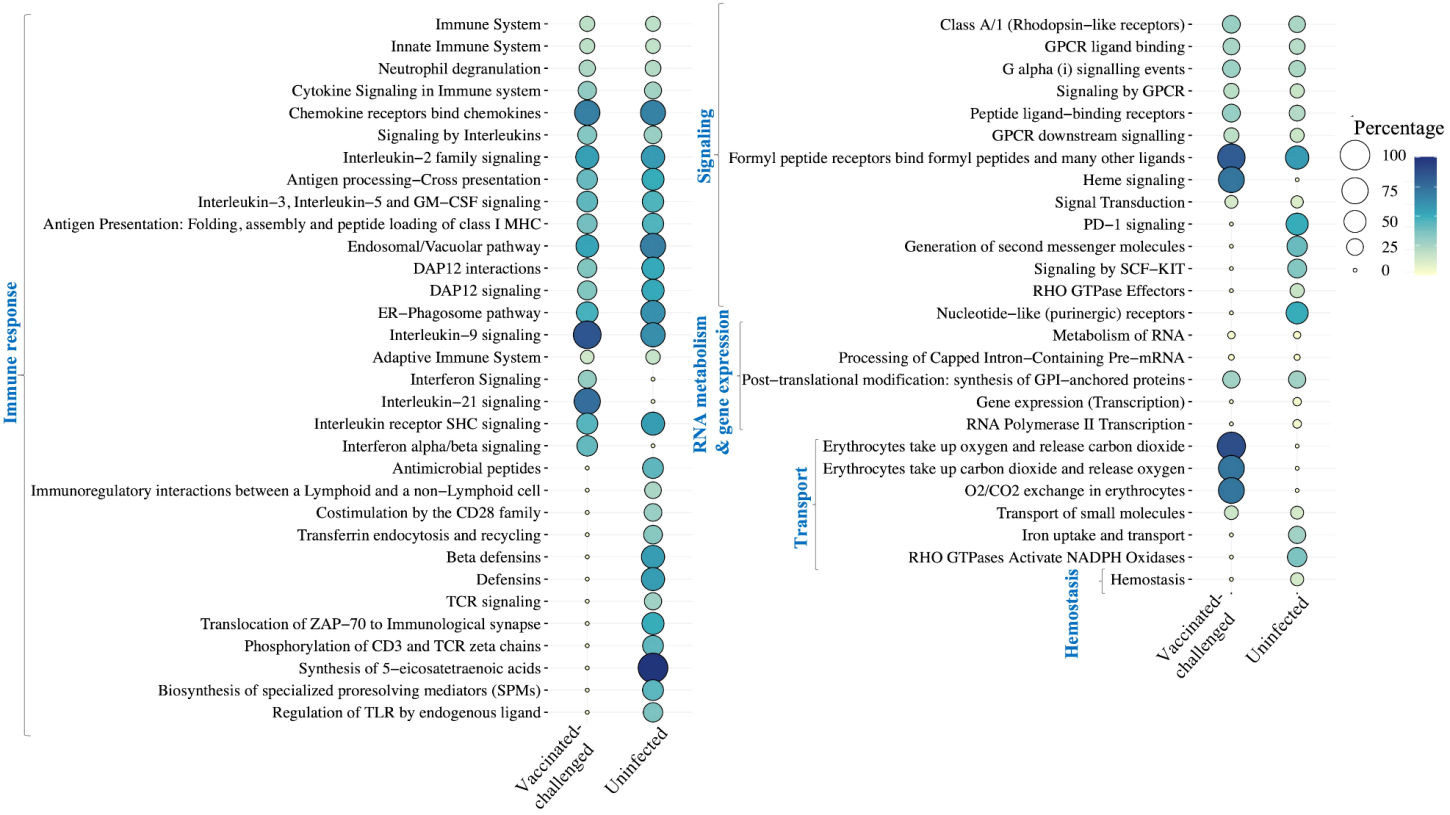
Reactome pathways upregulated in the non-vaccinated-challenged birds compared to the vaccinated-challenged or the uninfected birds at false discovery rate (FDR) <0.05. The pathways are arranged into categories based on functions, indicated in blue font on the left side. Percentage indicates the proportion of genes enriched in the pathway.

A total of 36 and 51 Reactome pathways were enriched with the genes upregulated in the non-vaccinated-challenged birds compared to the vaccinated-challenged and the uninfected birds, respectively (**Figure 6**). This included pathways related to immune response, signaling, RNA metabolism, gene expression, and transport, which were upregulated in the non-vaccinated-challenged birds compared to both the vaccinated-challenged and the uninfected birds as well as the pathway for “hemostasis”, upregulated solely in comparison with the uninfected birds. Of this, pathways involved in immune response formed the largest category that constituted 20/36 and 29/51 of the upregulated pathways in the non-vaccinated-challenged birds when compared to the vaccinated-challenged and the uninfected birds, respectively.

**Figure 7:**
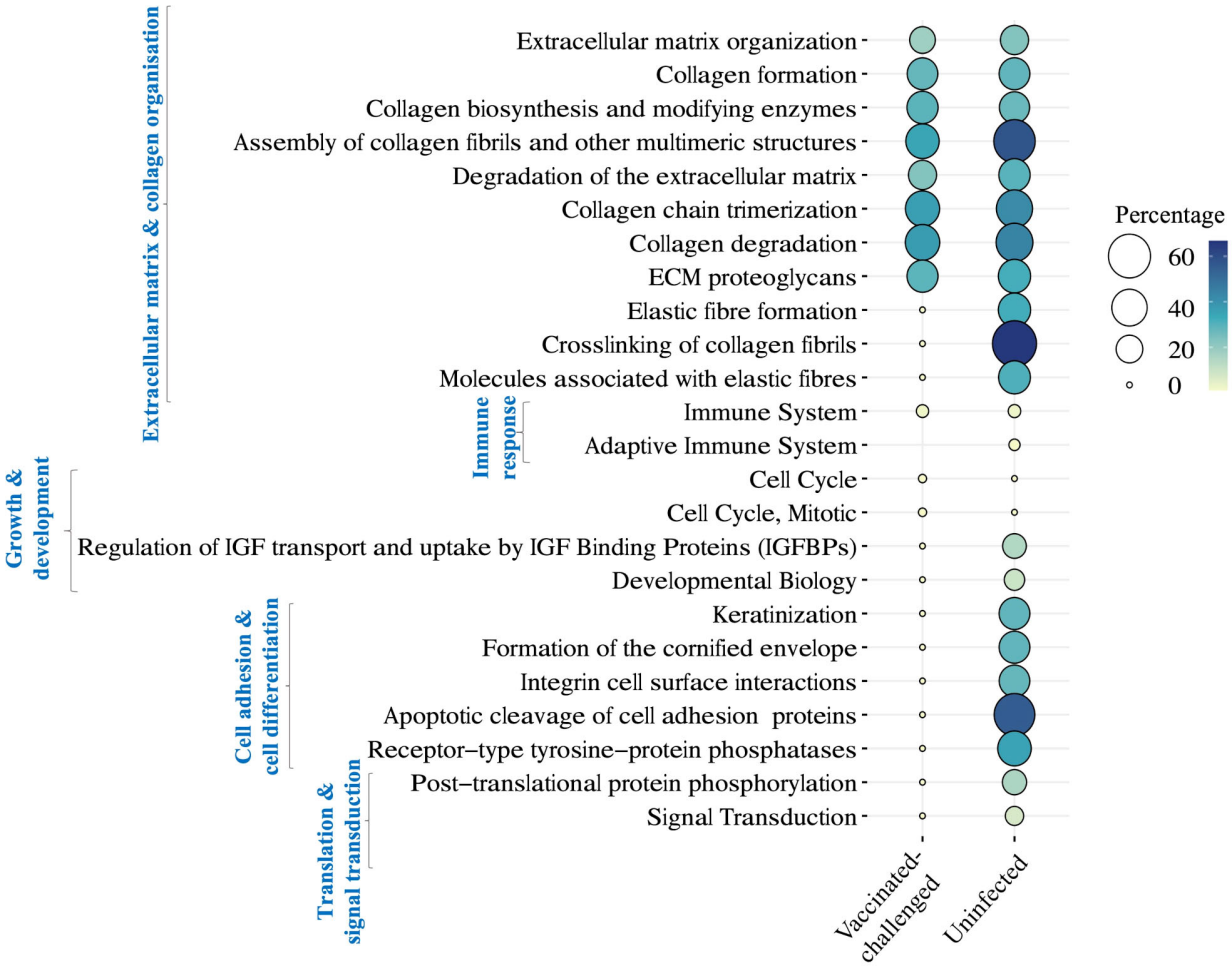
Reactome pathways downregulated in the non-vaccinated-challenged birds compared to the vaccinated-challenged or the uninfected birds at false discovery rate (FDR) <0.05. The pathways are arranged into categories based on functions, indicated in blue font on the left side. Percentage indicates the proportion of genes enriched in the pathway.

A total of 11 and 22 Reactome pathways were enriched with the genes downregulated in the non-vaccinated-challenged birds compared to the vaccinated-challenged and the uninfected birds, respectively (**Figure 7**). This included pathways related to extracellular matrix and collagen organization, immune response, and growth and development that were upregulated in the non-vaccinated-challenged birds compared to both the vaccinated-challenged and the uninfected birds as well as pathways related to cell adhesion and cell differentiation, translation, and signal transduction, downregulated solely in comparison with the uninfected birds. Of this, pathways involved in extracellular matrix and collagen organization formed the largest category that constituted 8/11 and 11/22 of the downregulated pathways in the non-vaccinated-challenged birds when compared to the vaccinated-challenged and the uninfected birds, respectively.

### 3.10 Immune response and ECM/structural organization formed the largest categories of the up- and downregulated protein classes, respectively, in the non-vaccinated-challenged birds

A total of 16 and 17 PANTHER protein classes were enriched with the genes upregulated in the non-vaccinated-challenged birds compared to the vaccinated-challenged and the uninfected birds, respectively (**Table 3**). These included protein classes with roles in defense and immune response, signal transduction, RNA metabolism, and/or gene expression that were upregulated in the non-vaccinated-challenged birds compared to both the vaccinated-challenged and the uninfected birds. Additionally, a class of enzyme was also upregulated in the non-vaccinated-challenged birds compared to the uninfected birds. The protein class enriched with the greatest number of upregulated genes per class in the non-vaccinated-challenged group was the “major histocompatibility complex protein” with 19/33 (57.58%) and 21/32 (65.63%) of the upregulated genes compared to the vaccinated-challenged and the uninfected birds, respectively.

**Table 3:** Protein classes upregulated in the non-vaccinated-challenged birds compared to the vaccinated-challenged or uninfected birds.

PANTHER protein class	Non-vaccinated-challenged vs.			
	Vaccinated-challenged		Uninfected	
	Percentage	FDR^*^	Percentage	FDR^*^
Immune response				
Defense/immunity protein	36.08	5.51E-44	39.74	1.70E-61
Immunoglobulin receptor superfamily	55.84	2.96E-48	60.81	7.50E-61
Major histocompatibility complex protein	57.58	2.85E-07	65.63	1.13E-09
Cytokine	48.15	1.58E-08	39.66	1.71E-07
Globin	15.39	2.70E-03	N	N
Chemokine	50.00	1.07E-02	N	N
Immunoglobulin	0.93	2.24E-02	N	N
Antimicrobial response protein	N	N	30.77	2.31E-02
RNA metabolism and/or gene expression				
Translational protein	0.62	2.94E-08	0.31	4.42E-08
RNA metabolism protein	3.38	1.34E-06	2.85	4.72E-06
RNA processing factor	0.90	3.27E-05	0.90	2.31E-04
Translation factor	0.93	7.01E-03	N	N
Zinc finger transcription factor	N	N	3.17	1.36E-02
DNA-binding transcription factor	N	N	5.17	2.64E-02
C_2_H_2_ zinc finger transcription factor	N	N	3.13	4.91E-02
Signaling				
Transmembrane signal receptor	15.81	2.55E-07	14.54	6.45E-08
G-protein coupled receptor	15.39	2.20E-03	14.62	4.06E-04
Intercellular signal molecule	16.51	7.93E-04	13.45	1.09E-02
Protein-binding activity modulator	13.62	2.16E-03	12.12	2.94E-03
Non-receptor tyrosine protein kinase	32.36	8.87E-03	29.41	1.14E-02
Enzyme				
Dehydrogenase	N	N	1.38	2.19E-02

* FDR, a false discovery rate <0.05 was considered significant. Percentage indicates the proportion of the genes enriched for each protein class.

N, not enriched.

Seven and 16 PANTHER protein classes were enriched with the genes downregulated (**Table 4**) in the non-vaccinated-challenged birds compared to the vaccinated-challenged and the uninfected birds, respectively. These included the protein classes for ECM and/or structural proteins, classes with role in cell adhesion, nucleic acid metabolism, translation, and immune response that were downregulated in the non-vaccinated-challenged birds compared to the other two groups. Additionally, a few classes of enzymes were also downregulated in the non-vaccinated-challenged birds compared to the uninfected birds. The protein classes enriched with the greatest number of downregulated genes per class in the non-vaccinated-challenged group were “extracellular matrix structural protein” with 16/80 (20%) and “intermediate filament binding protein” with 4/10 (40%) of the downregulated genes compared to the vaccinated-challenged and the uninfected birds, respectively.

**Table 4:** Protein classes downregulated in the non-vaccinated-challenged birds compared to the vaccinated-challenged or uninfected birds.

PANTHER protein class	Non-vaccinated-challenged vs.			
	Vaccinated-challenged		Uninfected	
	Percentage	FDR^*^	Percentage	FDR^*^
Nucleic acid metabolism and translation				
RNA metabolism protein	0.99	3.37E-04	0.71	1.82E-06
Translational protein	0.62	1.96E-02	0.31	4.34E-04
DNA metabolism protein	N	N	0.86	4.17E-02
Cell adhesion				
Cell adhesion molecule	10.64	8.57E-04	14.16	6.98E-06
Cadherin	N	N	14.43	7.60E-03
Extracellular matrix and/or structural proteins				
Extracellular matrix structural protein	20.00	1.06E-04	22.37	3.36E-05
Extracellular matrix protein	12.94	3.38E-04	14.55	1.38E-04
Intermediate filament	N	N	20.00	2.99E-02
Intermediate filament binding protein	N	N	40.00	3.37E-02
Gap junction	N	N	25.00	4.71E-02
Immune response				
Defense/immunity protein	N	N	0.85	4.30E-04
Immunoglobulin receptor superfamily	N	N	0.45	1.62E-02
Signaling				
Intercellular signal molecule	8.71	4.94E-03	10.53	4.90E-04
Enzymes				
Metabolite interconversion enzyme	N	N	2.75	1.25E-03
Transferase	N	N	2.08	1.91E-02
Growth factor				
Growth factor	12.00	2.89E-02	13.00	2.02E-02

* FDR, a false discovery rate <0.05 was considered significant. Percentage indicates the proportion of the genes enriched for each protein class.

N, not enriched.

## 4 Discussion

A comparative analysis of the mortality data, weight gain, clinical signs scores, tracheal gross and histopathology scores, and the qPCR results from tracheal swabs between the non-vaccinated-challenged and the vaccinated-challenged birds indicated that vaccination with ΔgG-ILTV provided effective protection against challenge with virulent ILTV. These results are consistent with those of a previous study that assessed the immunogenicity and pathogenicity of ΔgG-ILTV (5). The absence of any significant difference in tracheal transcriptome between the vaccinated-challenged and the uninfected birds, in contrast to the extensive differences between these groups and the non-vaccinated-challenged group at 4 to 5 dpc, provides further evidence of a high level of vaccine efficacy and helps to correlate differences in tracheal host gene transcription with the outcome of infection.

**Figure 8:**
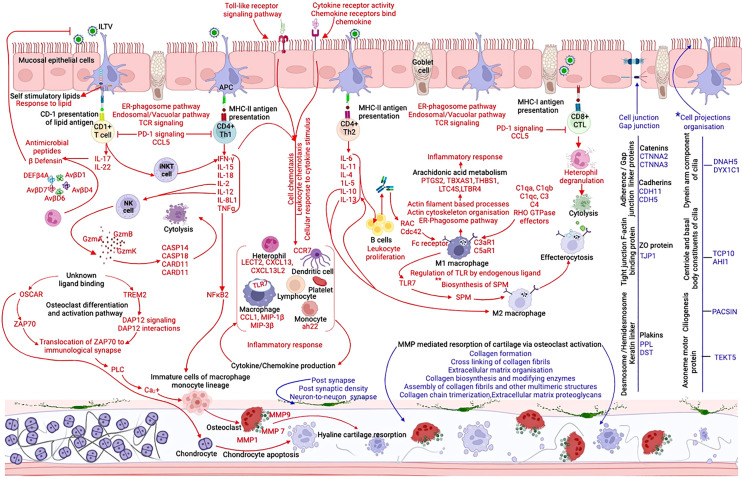
Schematic representation of the immune and inflammatory response in the trachea of non-vaccinated-challenged birds at 4 to 5 days post-challenge, created with BioRender.com. The arrows and texts in red indicate the upregulated and those in blue indicate the downregulated genes, gene ontologies, pathways, or protein classes in the non-vaccinated-challenged birds compared to the vaccinated-challenged and/or uninfected birds. *, differentially regulated only in comparison with the vaccinated-challenged group. **, differentially regulated only in comparison with the uninfected group.

Tracheal transcriptome data at 4 to 5 dpc (**Supplementary Tables S10**-**S13**) was used to collate an understanding of the molecular events pertaining to ILT infection, particularly immune response and the organization of structural components, that distinguished the non-vaccinated-challenged birds from the other two groups (**Figure 8**). The gene encoding *CCL20* was the only upregulated (downregulated in the non-vaccinated-challenged birds) chemokine in the vaccinated-challenged birds (**Supplementary Table S9**). Upregulation of this gene commonly with the uninfected group and its constitutive expression in mucosal tissues suggested that the chemokine potentially functioned in orchestrating homeostatic trafficking of leukocytes (46). This was consistent with the mild infiltration of inflammatory cells in the trachea of the vaccinated-challenged birds (**Figure 2F**), which aligned with their significantly lower tracheal pathology (12). Conversely, upregulation of serval TLRs (*TLR1B*, *TLR2A*, *TLR2B*, *TLR4*, *TLR7*, and *TLR15*) in the non-vaccinated-challenged birds (**Supplementary Table S8**) was consistent with the activation of several downstream signaling pathways that triggered diverse immune and inflammatory responses, thus exacerbating the tracheal pathology in these birds. Ligand-induced stimulation of *TLR2* and *TLR 4* has been shown to result in antiviral response to ILTV infection *in vitro* or *in ovo* (47–50), while the involvement of *TLR7* and *TLR15* has been demonstrated during Mark’s disease virus infection and *TLR1* during avian leukosis virus infections in chickens (51, 52) in previous studies.

Upregulation of the cathepsin-mediated “vacuolar pathway” as well as the cytosolic “ER-phagosome pathway” in the non-vaccinated-challenged birds (**Figure 6**) indicated the involvement of both TAP-independent and TAP- dependent mechanisms, respectively, for MHC-peptide antigen cross-presentation during ILTV infection (53, 54). Furthermore, upregulation of the genes encoding the CD1 molecules, *CD1b* and *CD1c* (gene synonyms *CD1.1*, *CD1.2*, *CD1A1*, and *CD1D*) (55, 56), also suggested the activation of the CD1-dependent lipid antigen presentation pathway (**Figure 8**) (57–59). The involvement of this pathway in the defense response to several bacterial and viral pathogens, including HSV-1, has been shown to be mediated through the activation of Th17 cytokines or through the activation of invariant natural killer T (iNKT) cells that facilitates an antiviral response through the enhanced production of Th1 cytokines like *IFN-γ* (60, 61). Activation of this pathway in the non-vaccinated-challenged birds is likely mediated via the interaction between virus-induced self-stimulatory lipids or lipopeptides (generated by cellular lipidation of viral proteins) and specific CD-1 restricted T cells (29, 57, 61–63). This predicted interaction was evidenced by the upregulation of BP terms indicating “response to lipid” in these birds (**Supplementary Figure S3**). In agreement with a previous study that demonstrated the destructive phagocytosis of ILTV-infected macrophages (64), the current study detected evidence of both type I antibody-mediated and type II complement-mediated opsonophagocytosis (65) in the non-vaccinated-challenged birds (**Figure 8**). This was substantiated by the upregulation of genes encoding several Rho-GTPase-activating/associated proteins, complement receptors, and Fc-fragment receptors and the enrichment of BP terms indicating actin cytoskeleton remodeling in these birds (**Supplementary Figure S3**) (66).

The upregulated CD molecules in the non-vaccinated-challenged birds (**Supplementary Table S8**) indicated diverse cell types populating their trachea, consistent with the histopathological findings (**Figures 2C, D**). This aligned with the upregulation of several cytokine- and chemokine-encoding genes (**Supplementary Table S8**) and the upregulated gene ontologies indicating immune cell migration (**Supplementary Figures S3**, **S6**). The upregulated chemokine- and cytokine-encoding genes in these birds included the -C-X-C family of chemokines and *LECT2* that are chemotactic for heterophils/neutrophils (67, 68), several -C-C family of chemokine that are chemotactic for monocytes and T cells (68), and *ah221*, belonging to the monocyte chemotactic family (69), among the many others. The presence of dendritic cells was indicated by the upregulation of *CCR7*, a chicken dendritic cell marker (70), while the upregulation of macrophage inflammatory proteins, *MIP-1b* (*CCL4*) and *MIP-3b* (*CCL19*), was consistent with the recruitment of macrophages (71–73).

It could be hypothesized that the downregulation of *IL1R1* promoted the upregulation of *IL-1β* in the non-vaccinated-challenged birds as *IL1R1*–*IL-1β* interactions result in negative feedback, reducing the *IL-1β* levels (74). Similarly, downregulation of the gene encoding *IL17REL*, a negative regulator of *IL-17* receptors (75), might have enhanced the expression of the *IL-17A*- and *IL-17F*-encoding genes in these birds, consequently promoting the production of several proinflammatory cytokines, recruitment of heterophils, and the production of antimicrobial peptides such as β-defensins (76, 77) (**Figure 6**). Among the 14 avian β-defensins identified to date (78), five were upregulated in the non-vaccinated-challenged birds (**Figure 8**). Their role in antiviral immunity to ILTV has not been demonstrated yet. Nevertheless, multiple antiviral mechanisms demonstrated for these innate immune components against Marek’s disease virus (MDV), chicken infectious bronchitis virus (IBV), and Newcastle disease virus (NDV) in chickens (79–83) warrant investigation of their role during ILTV infection.

The results of cytokine and chemokine transcription in the non-vaccinated-challenged birds were largely in agreement with Vagnozzi et al. and the *in vivo* component of the investigation conducted by Coppo et al. (12, 84). Importantly, the absence of *IFN-β*, but *IFN-γ* gene upregulation in the nonvaccinated- challenged birds underscores the finding that ILTV infection interferes with *IFN-β* gene transcription (84). Although there was no upregulation of the gene encoding *IFN-α* in these birds, upregulation of genes for several interferon α-inducible proteins and the upregulated pathway for “interferon α/β signaling” suggested its activation. There are very limited studies that explored the functions of *IFN-κL1* and *IFN-ω1* in chickens (85). The upregulation of these genes in the non-vaccinated-challenged birds during the peak of infection indicates that they likely have a role in immune response to ILTV infection in chickens. Genes encoding the anti-inflammatory cytokines *IL-10* and *IL-13*, upregulated in the non-vaccinated-challenged birds, were also in agreement with Vagnozzi et al. (2018), who noticed their peak upregulation at 5 dpi, parallel to the severe pathological changes in the trachea (84). These chemokines were suggested to have a role in dampening the inflammatory responses through M2 macrophage polarization (**Figure 8**), and hence their upregulation suggests the onset of a transition from proinflammatory phase to tracheal regenerative phase (84). In addition to this, the pathway upregulated for “PD-1 signaling”, which inhibits T cell activation, proliferation, and survival (86), in combination with two other upregulated pathways involved in the production of specialized pro-resolving mediators (SPM) (**Figures 6**, **8**), which promotes M2 macrophage polarization and effecterocytosis, consequently dampening the inflammatory responses (87–89) (**Figure 8**), and underpins the notion of tissue restoration in these birds.

As observed in a few recent ILTV studies (71, 90), the non-vaccinated-challenged birds in the current study also displayed loss of or damage to the cilia on tracheal epithelial cells (**Figures 1D**, **2C**). Consistent with these observations, the biological process of “cell projections organization” (**Supplementary Figure S9**) as well as several genes (*DNAH5*, *DYX1C1*, *TCP10*, *AHI1*, *PACSINs*, and *TEKT5*) involved in ciliogenesis, cilium assembly, and ciliary motility (91–96) were downregulated in these birds (**Figure 8**). This indicated that damage to ciliary components during ILTV infection is not solely a consequence of viral replication in mucosal epithelial cells but, rather, is regulated at the transcriptome level, thus confirming suggestions from an earlier study (12). Due to the significant role that cilia play in the mucociliary clearance of particles including viruses in respiratory epithelium, impairment of their function indicates a compromise to innate immune mechanisms in these birds. There were also indications of damage to the components of the extracellular matrix and cartilaginous structures in the trachea of the non-vaccinated-challenged birds (**Figure 8**). Several alleles of the gene encoding the immunoglobulin receptor *OSCAR*, upregulated in the non-vaccinated-challenged birds (**Supplementary Table S8**), are directly correlated to the destruction of chondrocytes via the induction of chondrocyte apoptosis in cartilages (97). Furthermore, the other upregulated genes encoding *TREM-2*, *ZAP70*, and *PLC*, together with *OSCAR*, induce the Ca^2+^ influx necessary for the differentiation and maturation of osteoclasts, which further mediates cartilage resorption/ECM and collagen degradation through the expression of MMPs (also upregulated) (98, 99). These predicted effects also aligned with the upregulation of signaling pathways for *DAP12* and *ZAP-70* molecules, positive regulation of cytosolic calcium ion concentrations, and the simultaneous downregulation of several pathways for ECM and collagen organization and synthesis (**Figures 6**–**8**) in non-vaccinated-challenged birds. Activation of osteoclast by *NF-kB* is yet another possible pathway that culminated in these potential structural modifications (99) (**Figure 8**). Previous studies have also reported the upregulation of MMPs following the infection of chicken embryo lung cells with wild-type ILTV (100) and downregulation of several classes of collagen in chickens immunized with a CEO ILT vaccine that associated these changes to a potential compromise to tracheal functions and structure (101). Nevertheless, immunostaining using osteoclast-specific and chondrocyte-specific markers is required to confirm this hypothesis, as these changes were not obvious upon H&E staining of tracheal tissues in the current study (**Supplementary Figure S15**).

Downregulation of the “gap junction” class of proteins (**Table 4**) and the CC term “cell junction” (**Figure 5**) enriched with several barrier molecules (BMs) in the non-vaccinated-challenged birds suggested impairment to junction complexes (**Figure 8**). Downregulated BMs included catenins (*CTNNA2* and *CTNNA3*), cadherins (*CDH13*, *CDH11*, and *CDH5*), plakins (*PPL* and *DST*), and the tight junction ZO protein *TJP1* (102–104). These BMs play integral roles in the formation of various junction complexes, including tight junctions (TJs), adherens junctions (AJs), and desmosomes (105, 106) (**Figure 8**). Disruption of junction complexes in these birds possibly enhanced the trans-endothelial migration of leukocytes (107) and elevated the chemokine milieu at the site of infection, which aggravated the tracheal pathology (108). This may also have facilitated viral spread outside of the tracheal mucosae (109) of these birds as seen in HSV-1 infection (110) and as evidenced by the detection of ILTV in a range of extra-respiratory and neural tissues reported in numerous previous studies (11, 111–115). Aligning with these findings, the current study demonstrated downregulation of several GO terms related to neuron generation and synapse formation (**Figures 5**, **8**; **Supplementary Figures S9**, **S11**, **S12**, **S14**) in the non-vaccinated-challenged birds. These results, in combination, could reflect ILTV infection of neurons innervating tracheal tissues (116), consequently inducing the transcriptional modifications of these genes. While it remains unclear whether ILTV infects peripheral nervous structures within the trachea, previous research has shown evidence of latent infection in tracheal tissues (115) as well as in sensory neurons of the trigeminal ganglia (11, 115). Infection of the peripheral neural ganglia innervating the trachea would also be consistent with the impairment of cell junction components at the epithelium, while the persistence of virulent ILTV in non-vaccinated-challenged birds may have prolonged the window of opportunity for neuronal infection in these birds compared to the vaccinated-challenged birds (11). Further studies aimed at detecting and quantifying the virus within neuronal tissues and trigeminal ganglia following ILTV infection of both ΔgG-ILTV vaccinated and non-vaccinated birds are required to validate these findings.

Taking these results in combination, the differences in tracheal transcriptome between the ΔgG-ILTV vaccinated and non-vaccinated-challenged birds aligned with the clinical outcomes observed following challenge with virulent ILTV in these groups of birds. A heightened immunological response and a prolonged viral persistence, together with the pathological changes in mucosa, junctional complexes, and cytoskeletal structures, that impaired ciliary and neuronal functions were associated with virulent ILTV infection in the non-vaccinated-challenged birds. In contrast, vaccination appeared to result in protection from damage to the cellular components and significantly reduced the immune and inflammatory responses associated with ILTV pathology in the vaccinated-challenged birds so that the transcriptomic and clinical profiles of these vaccinated birds after challenge were a little different from those of uninfected birds. It appears that the vaccinated birds attained immune homeostasis shortly after ILTV challenge. Future studies aimed at evaluating the immune responses associated with the protection offered by the ΔgG-ILTV vaccine should focus on assessing the early stages of transcriptional changes after ILTV vaccination and again soon after challenge. These studies have the potential to unveil the specific markers that underline the immune protection triggered by ILTV exposure, whether through vaccination or natural infection. Given the critical role of cell-mediated immune response in ILT disease protection and ILTV-induced pathology, studies investigating tracheal cellular dynamics after vaccination and after virulent ILTV challenge are also warranted.

## Data Availability

The datasets presented in this study can be found online at NCBI, under the accession number: PRJNA1100627 (SRA).
