# Supplementary figures and images for: Clinical assessment and transcriptome analysis of host immune responses in a vaccination-challenge study using a glycoprotein G deletion mutant vaccine strain of infectious laryngotracheitis virus
Source: Front Immunol. 2025 Jan 24;15:1458218. doi: 10.3389/fimmu.2024.1458218 (PMC11802539; doi:10.3389/fimmu.2024.1458218)

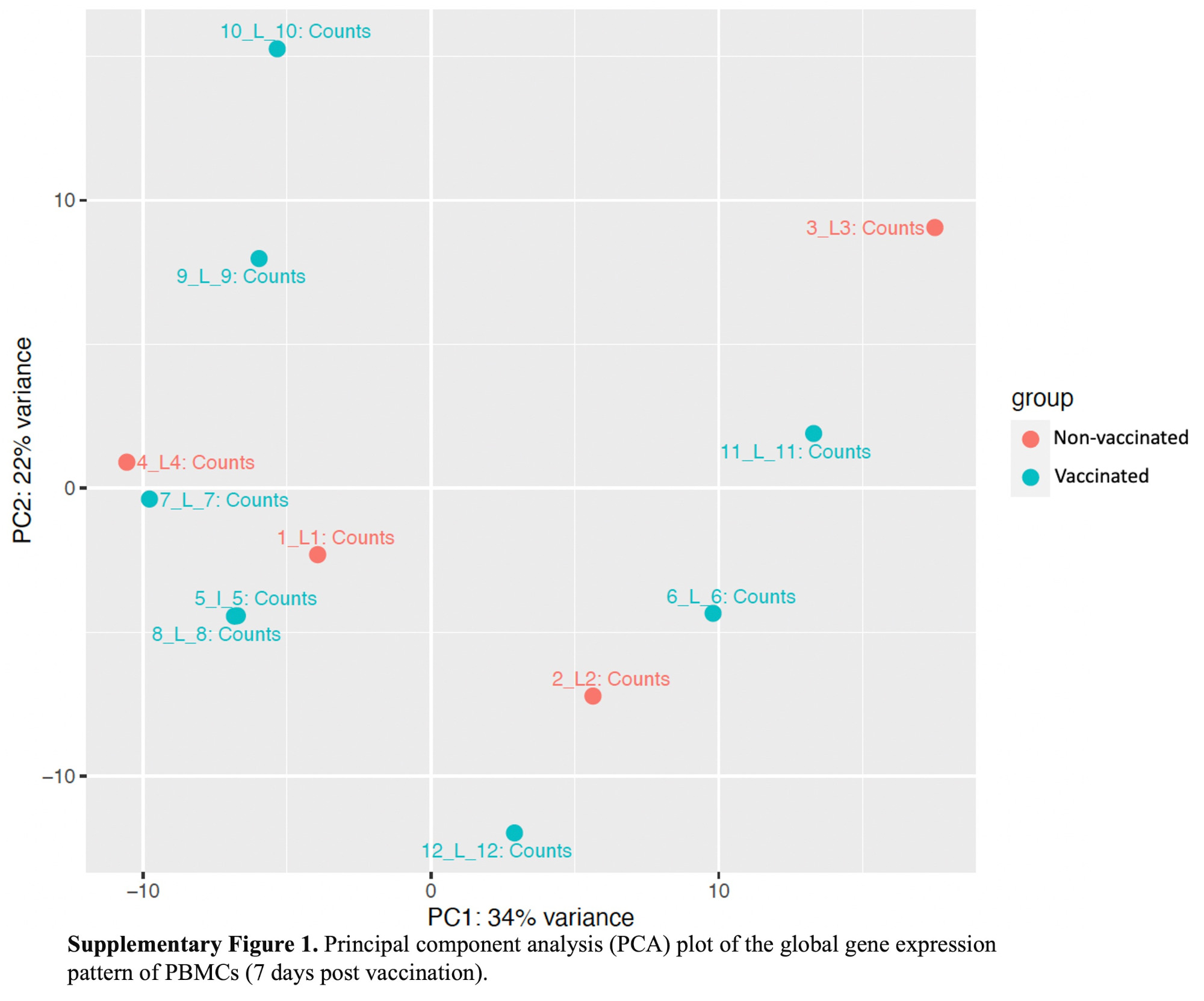

Supplement: Supplementary file 1 [file Image1.jpeg]

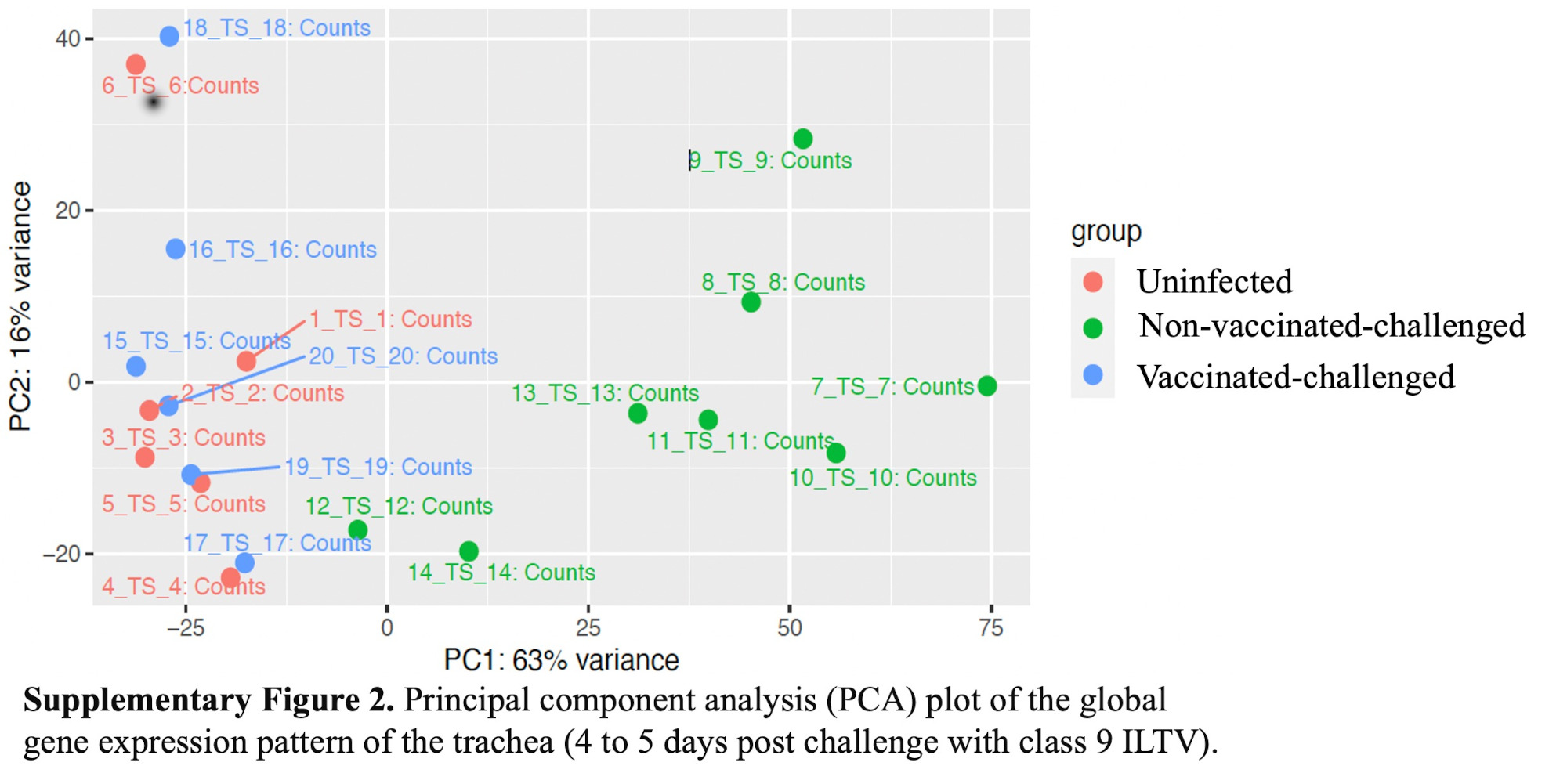

Supplement: Supplementary file 2 [file Image2.jpeg]

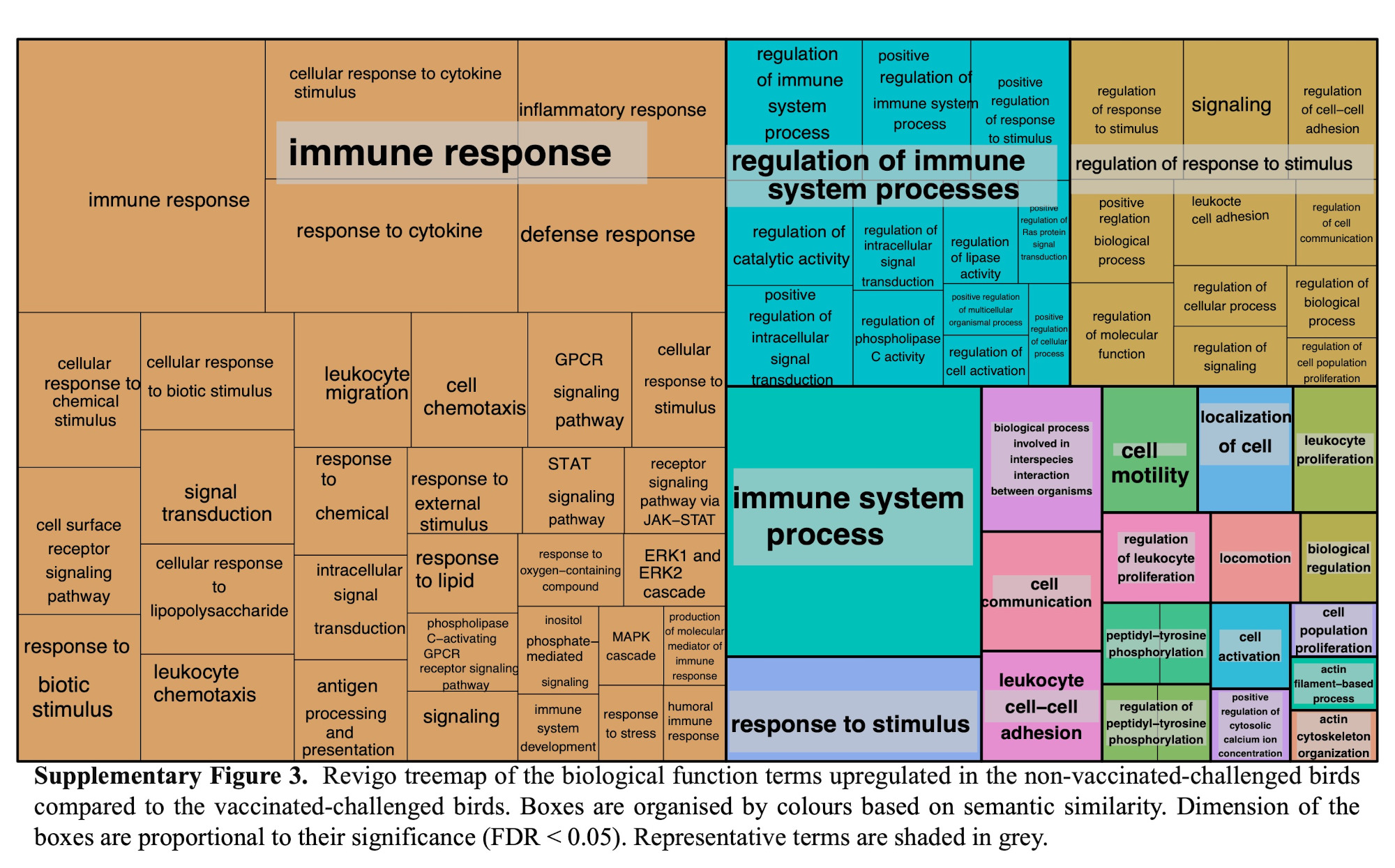

Supplement: Supplementary file 3 [file Image3.jpeg]

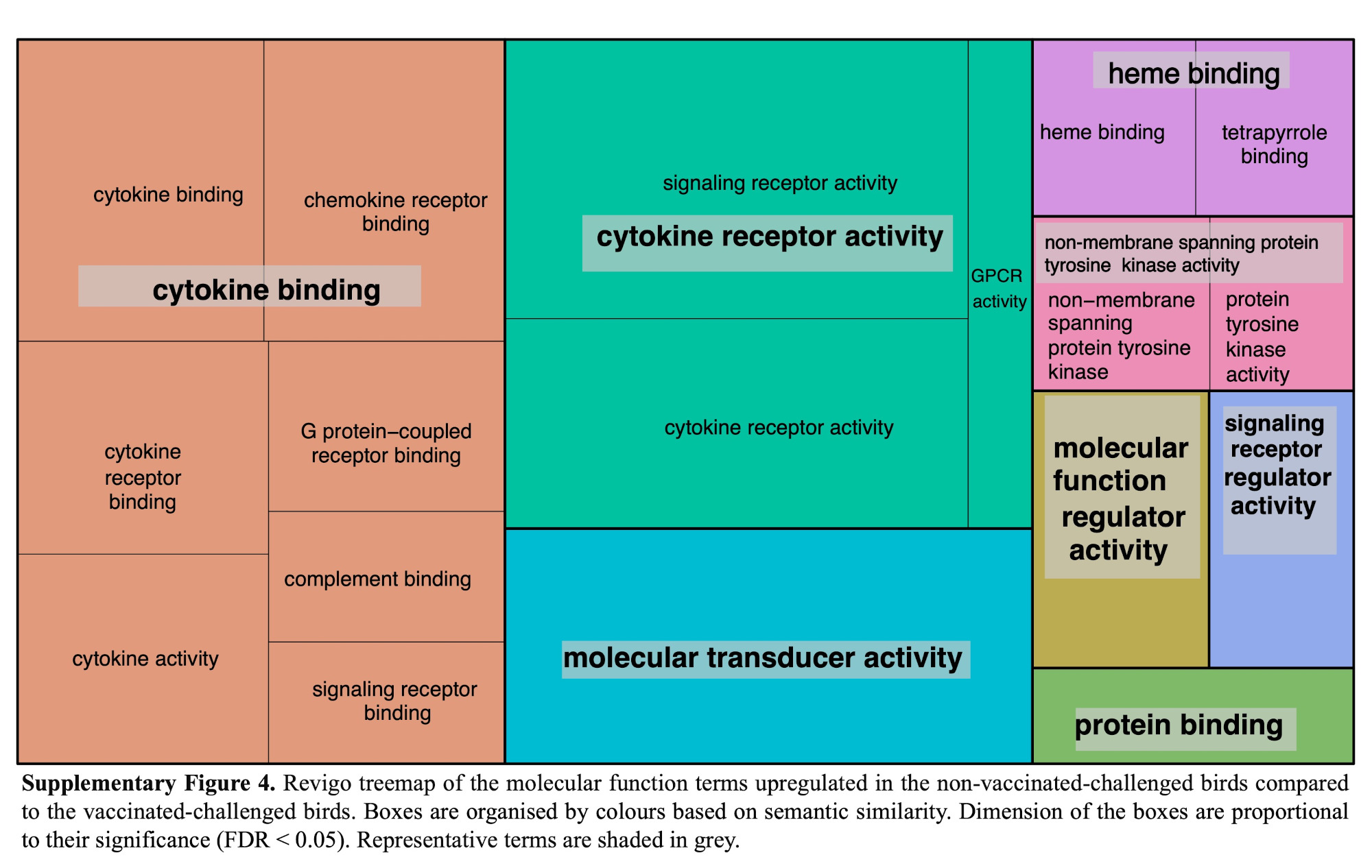

Supplement: Supplementary file 4 [file Image4.jpeg]

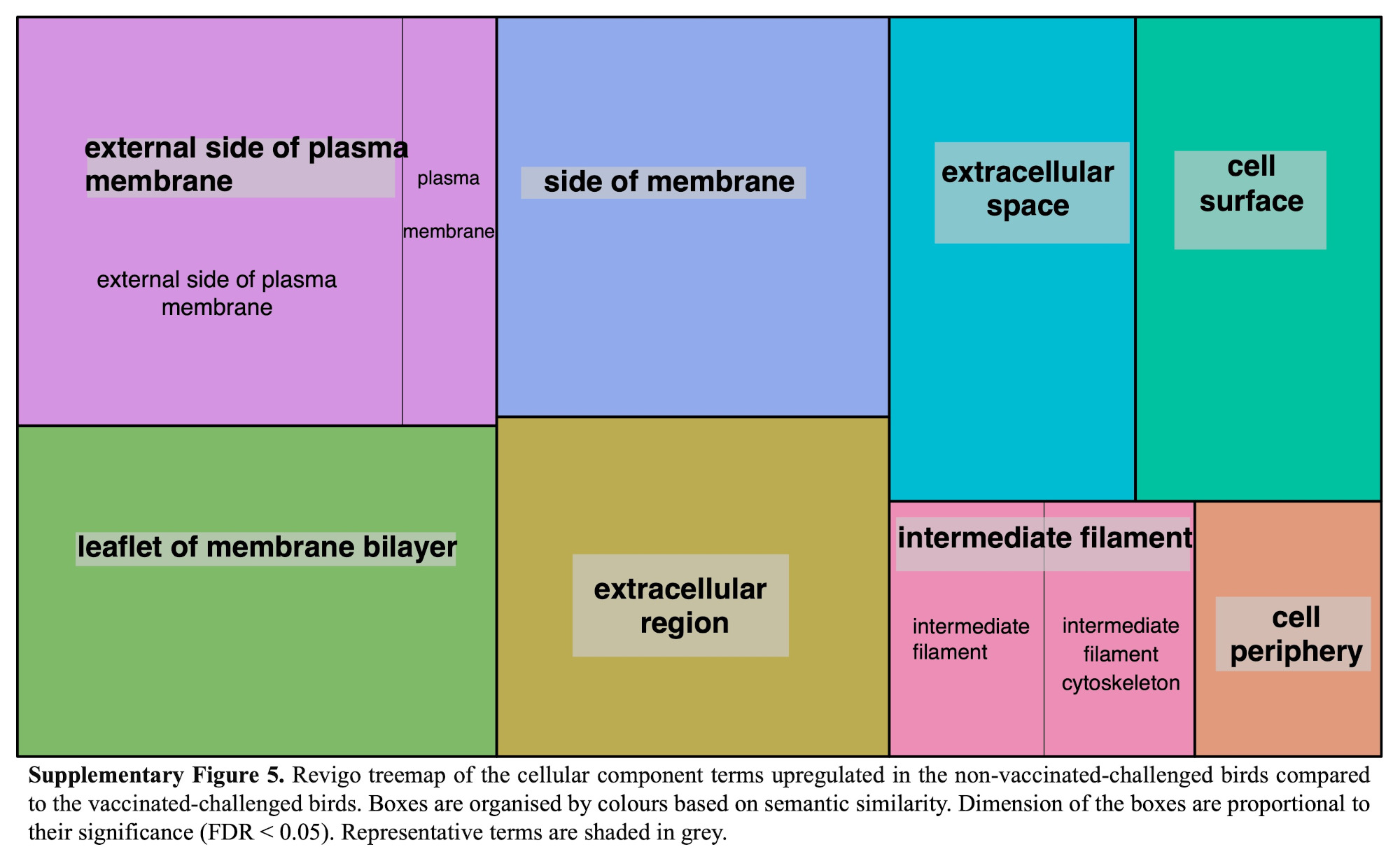

Supplement: Supplementary file 5 [file Image5.jpeg]

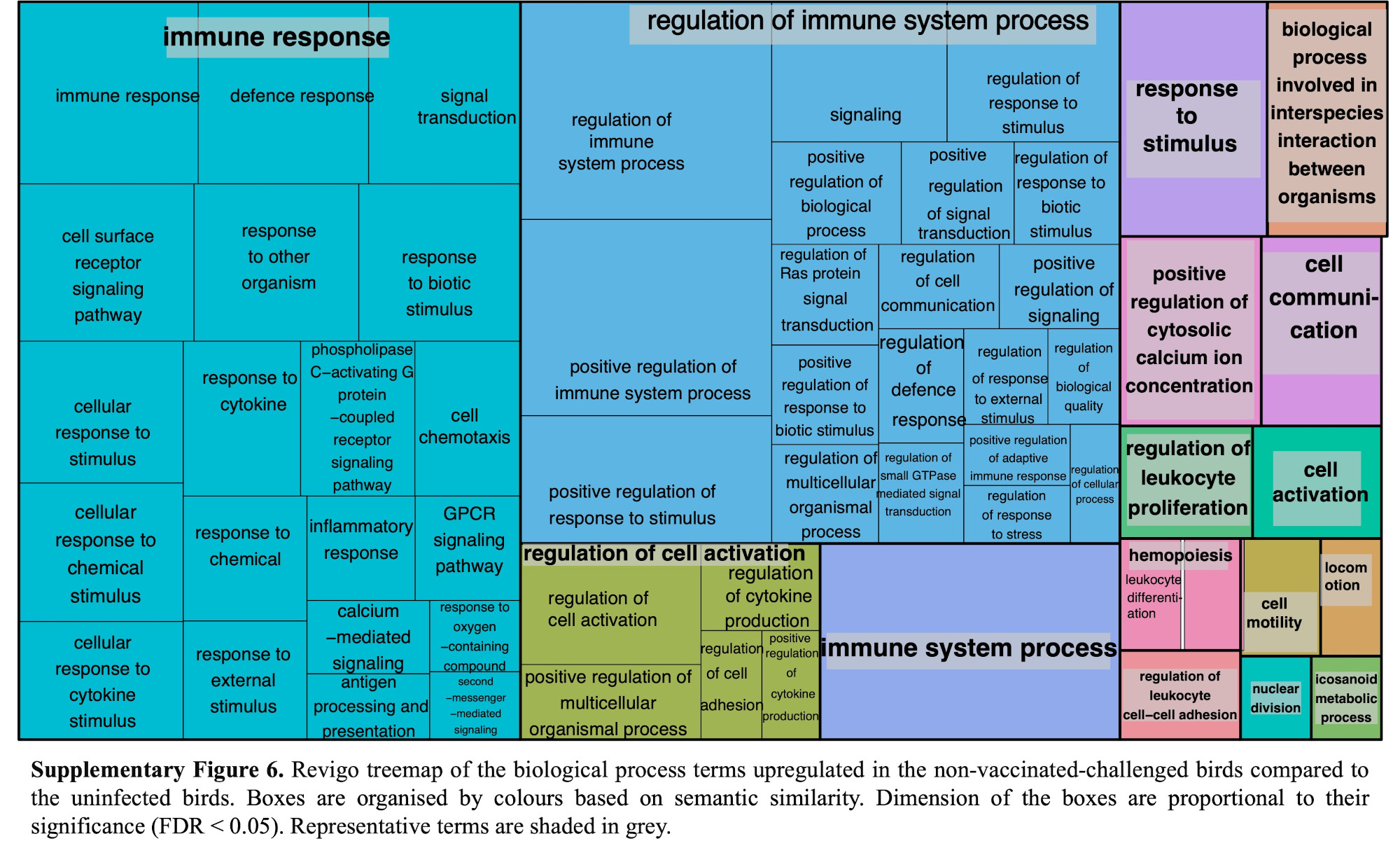

Supplement: Supplementary file 6 [file Image6.jpeg]

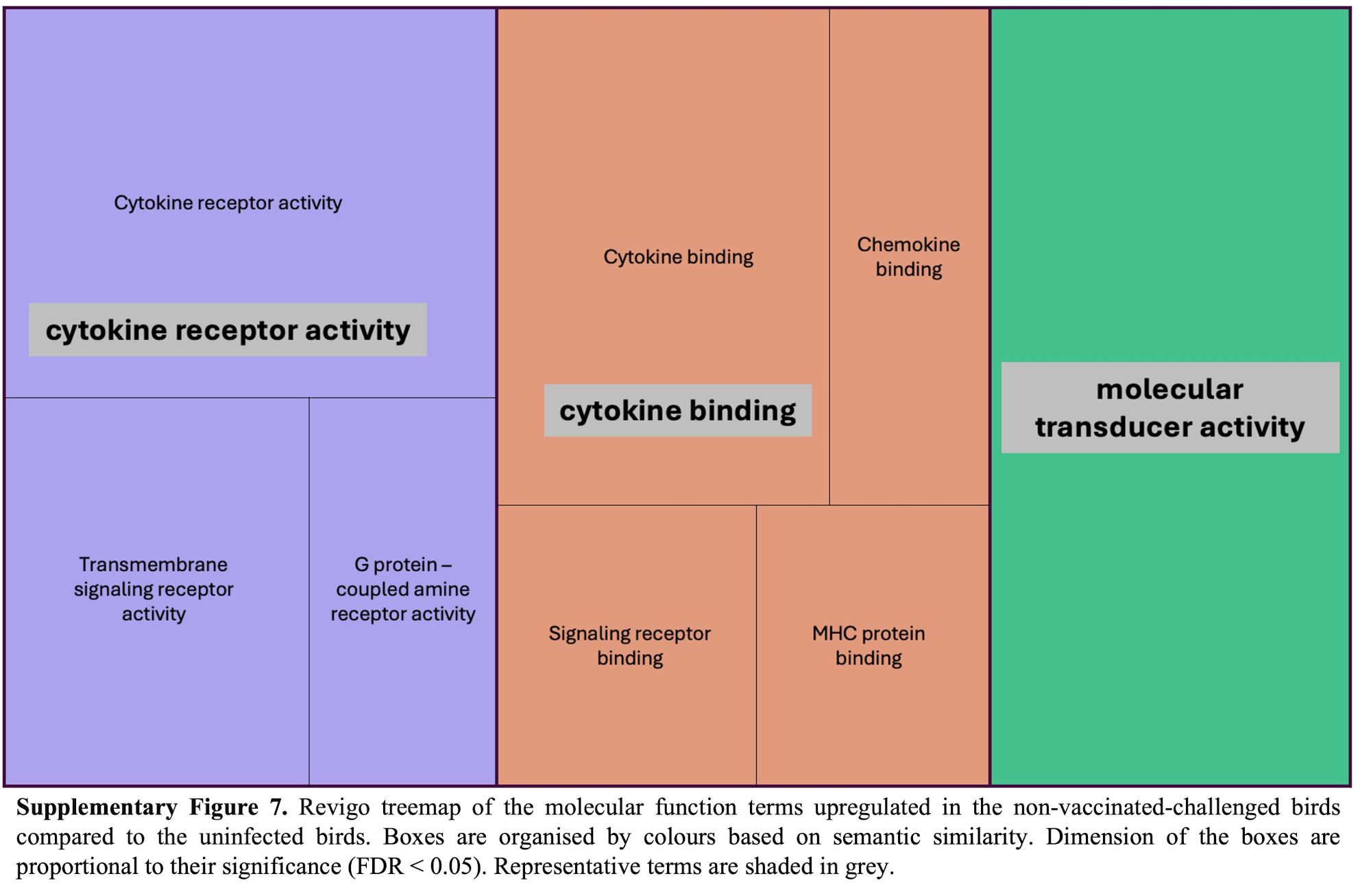

Supplement: Supplementary file 7 [file Image7.jpeg]

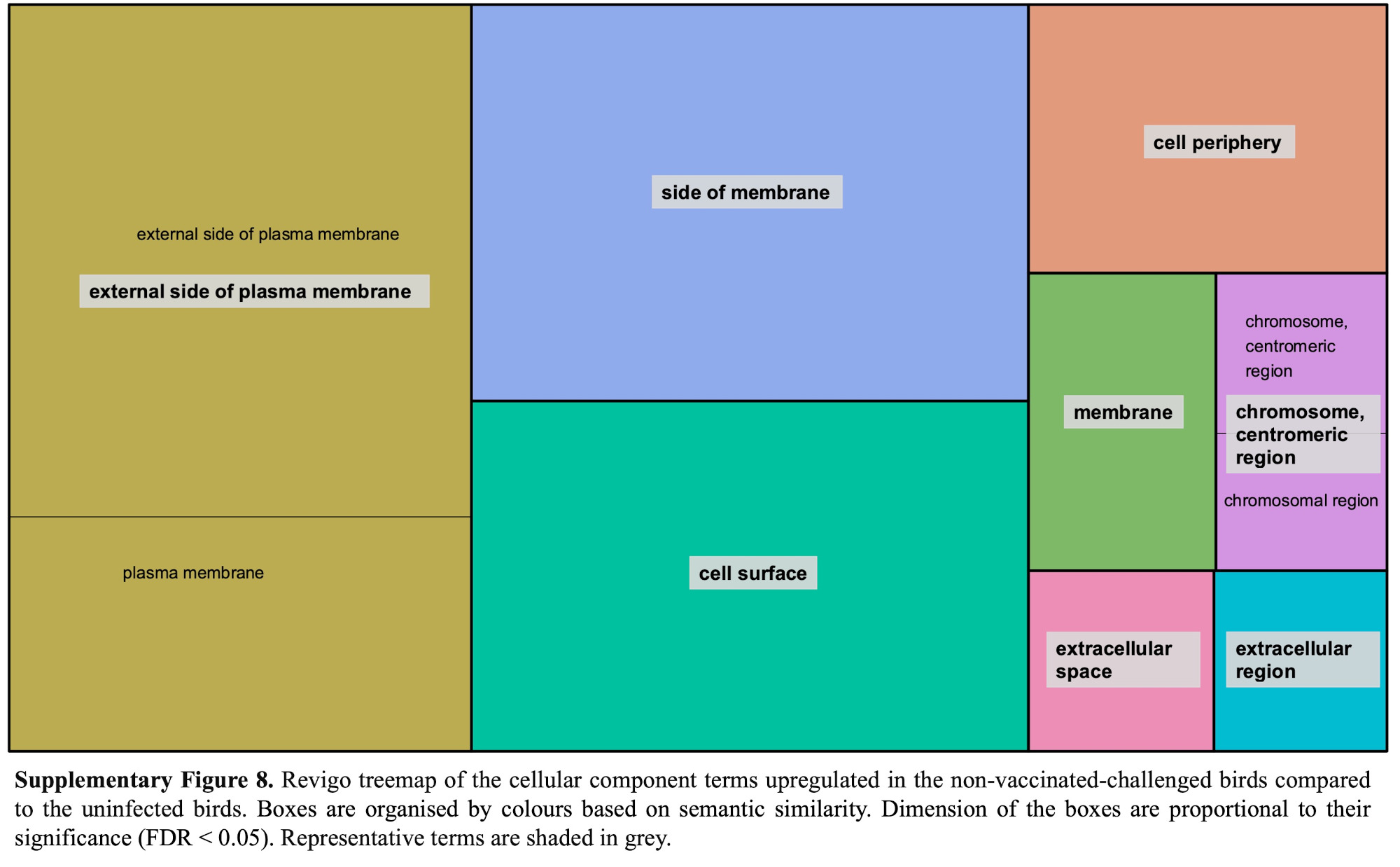

Supplement: Supplementary file 8 [file Image8.jpeg]

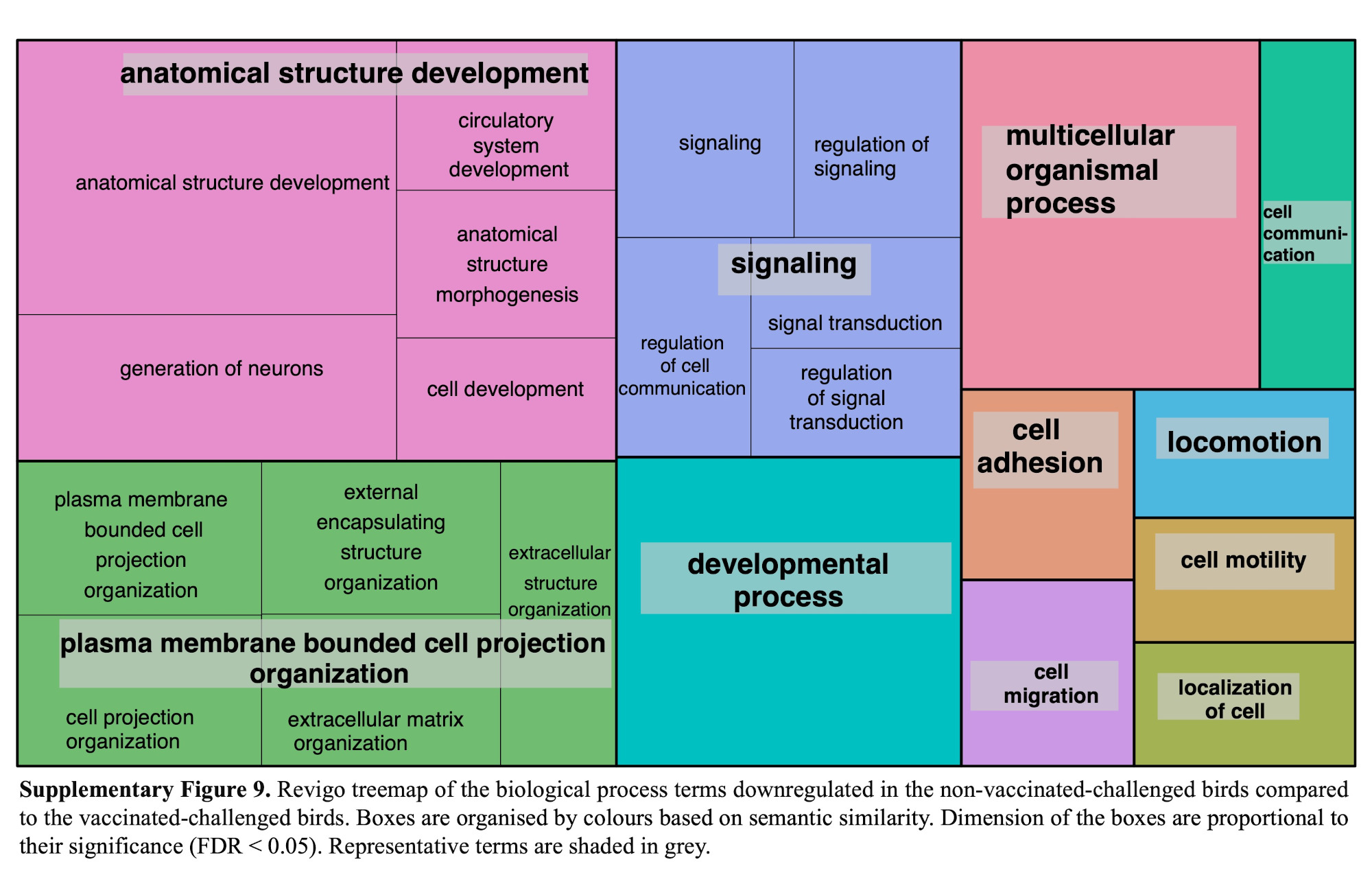

Supplement: Supplementary file 9 [file Image9.jpeg]

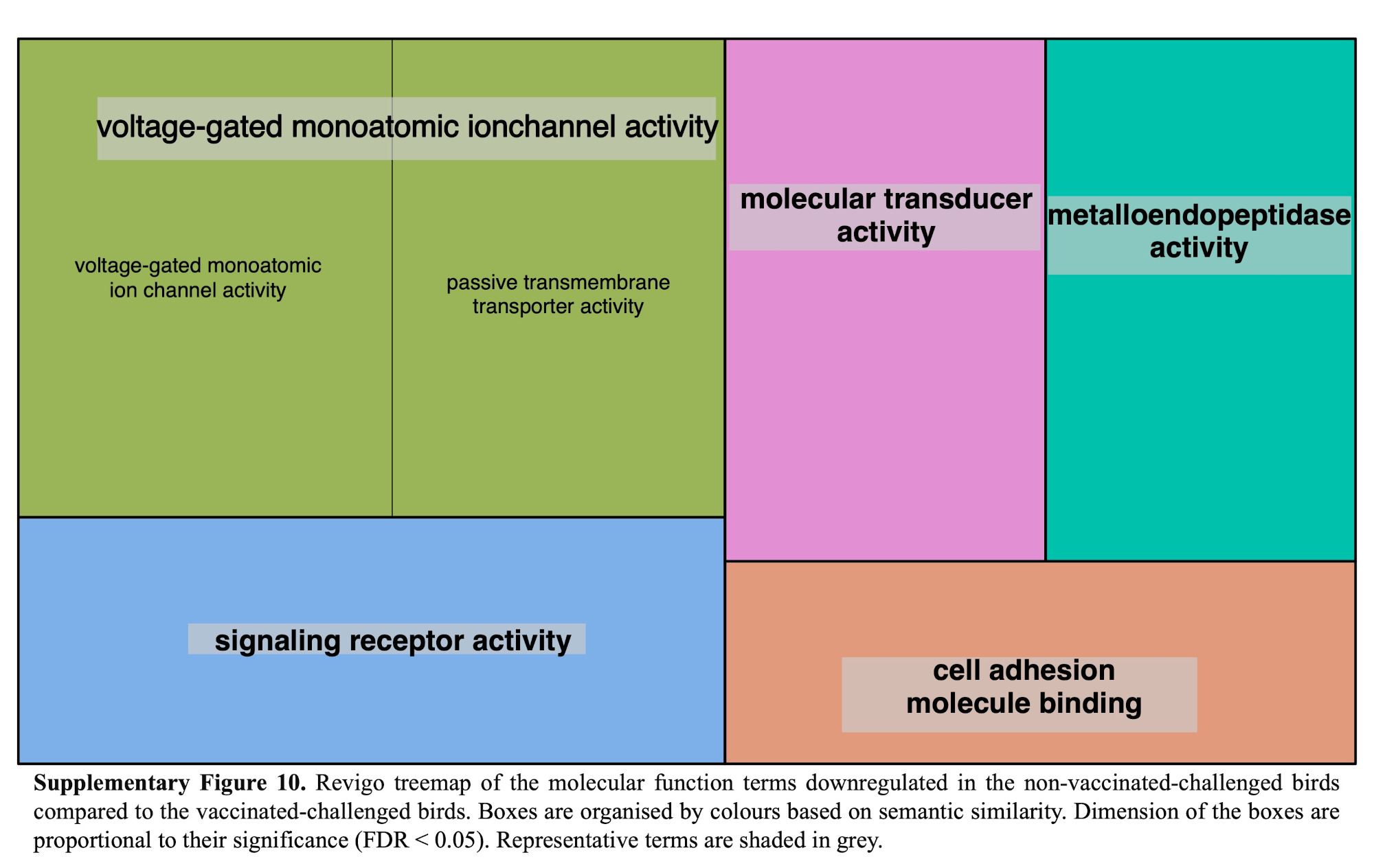

Supplement: Supplementary file 10 [file Image10.jpeg]

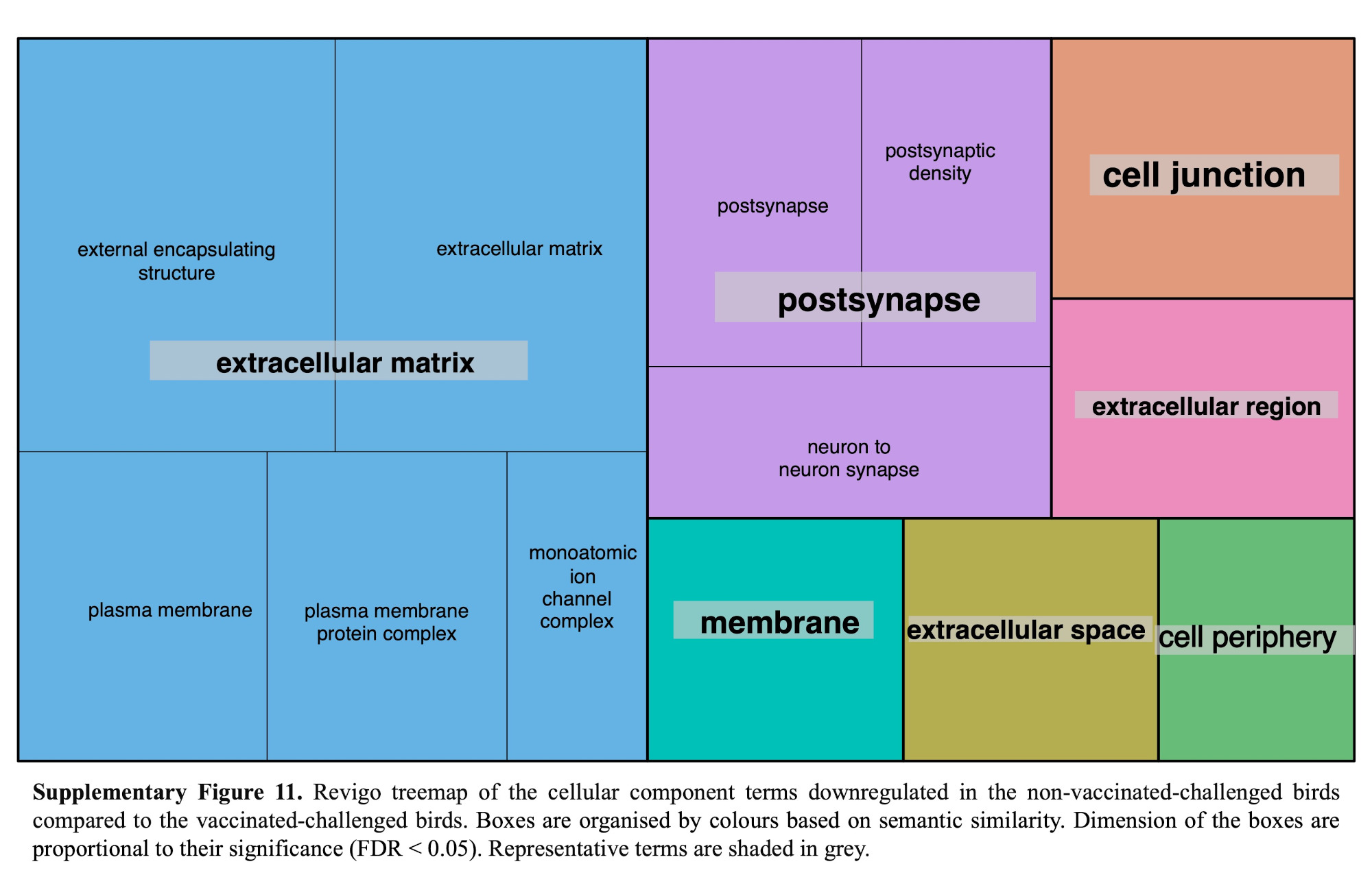

Supplement: Supplementary file 11 [file Image11.jpeg]

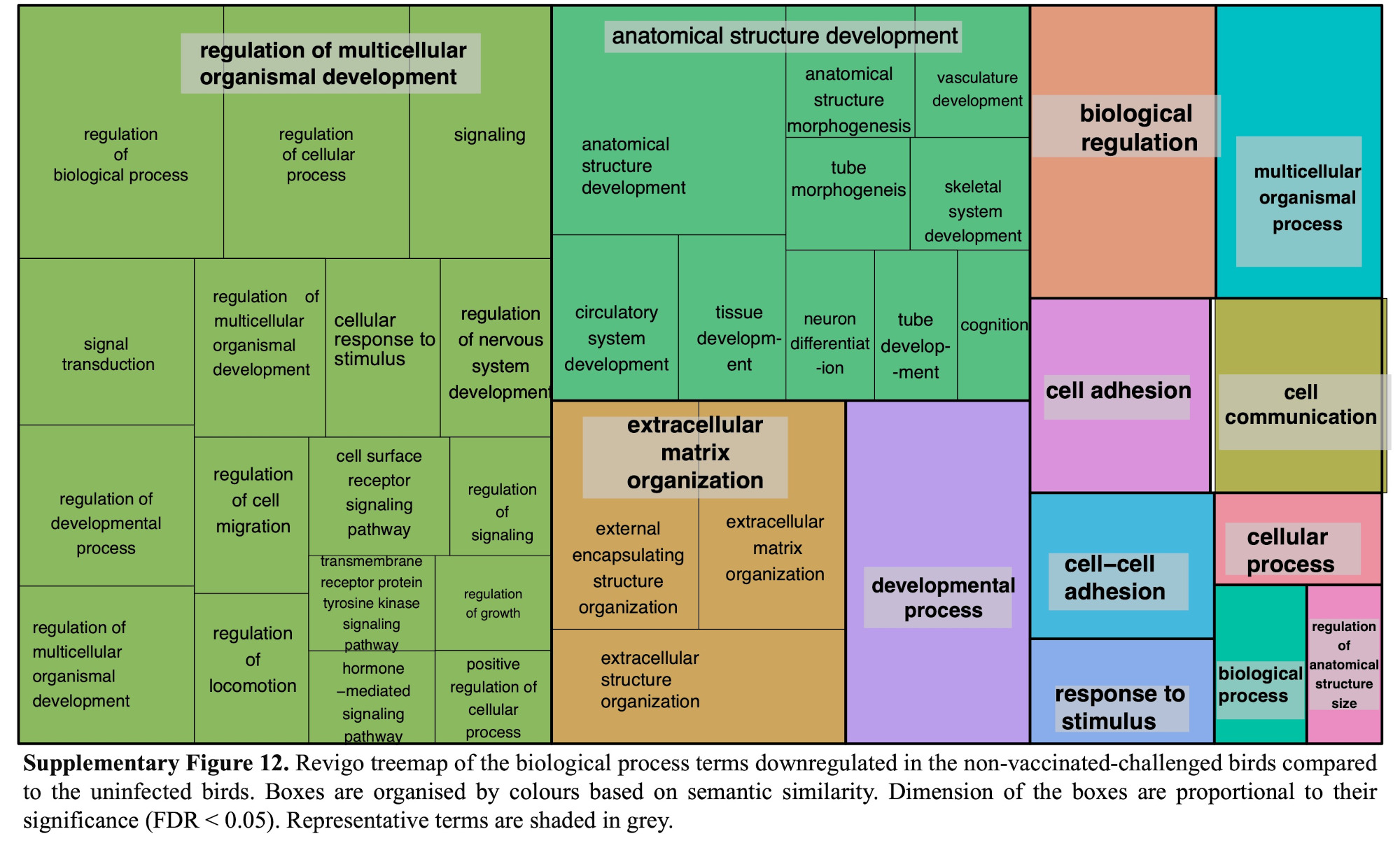

Supplement: Supplementary file 12 [file Image12.jpeg]

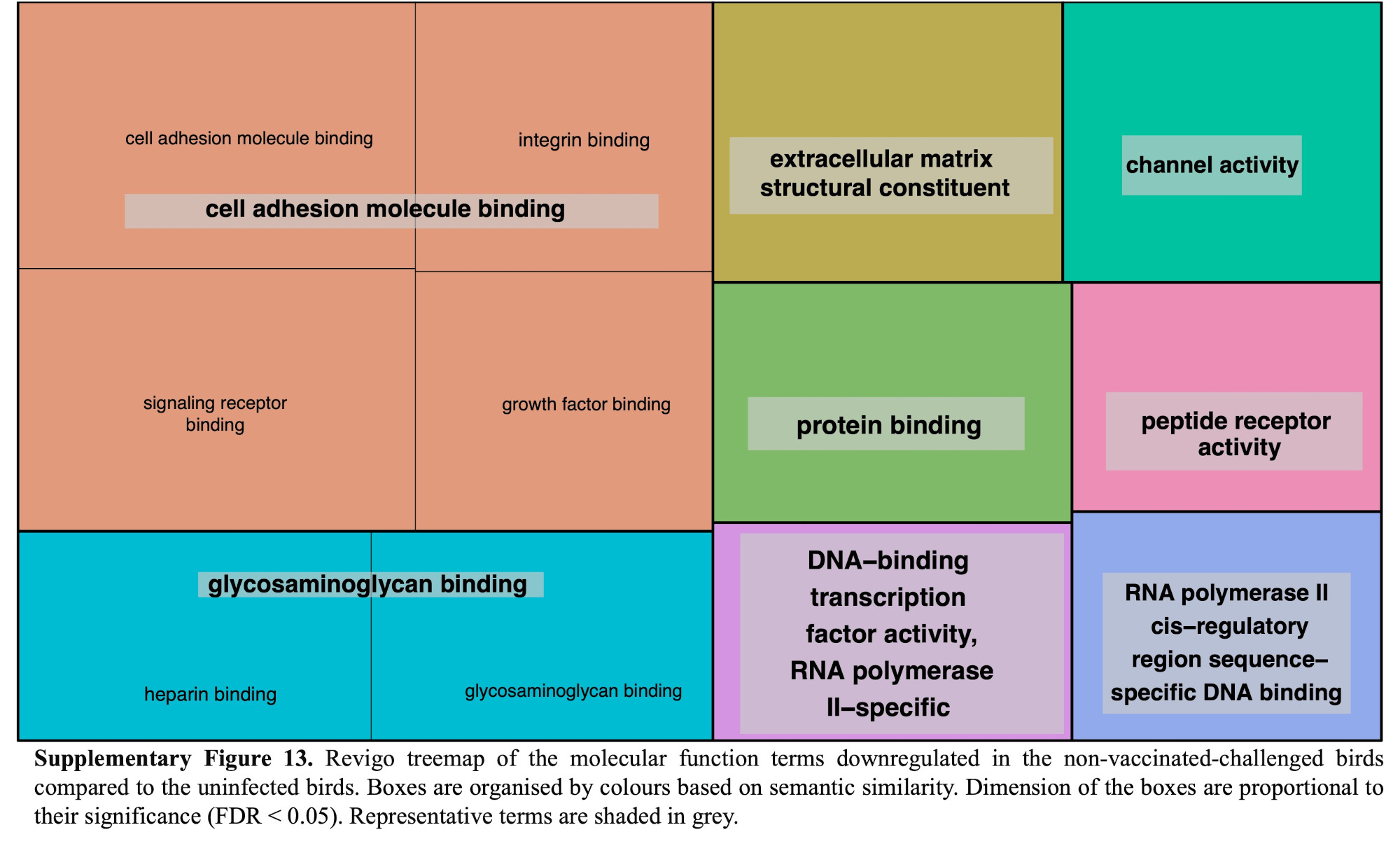

Supplement: Supplementary file 13 [file Image13.jpeg]

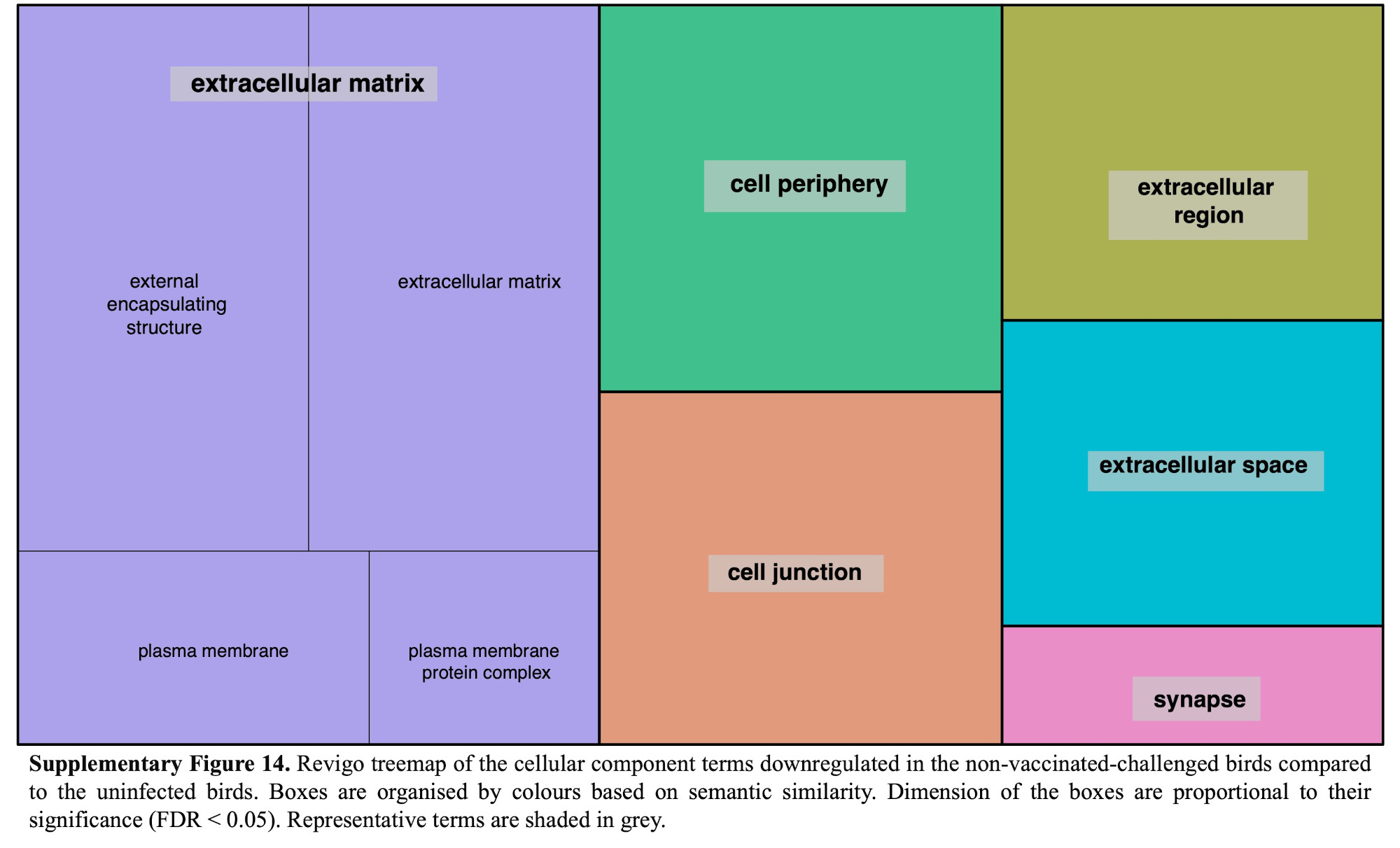

Supplement: Supplementary file 14 [file Image14.jpeg]

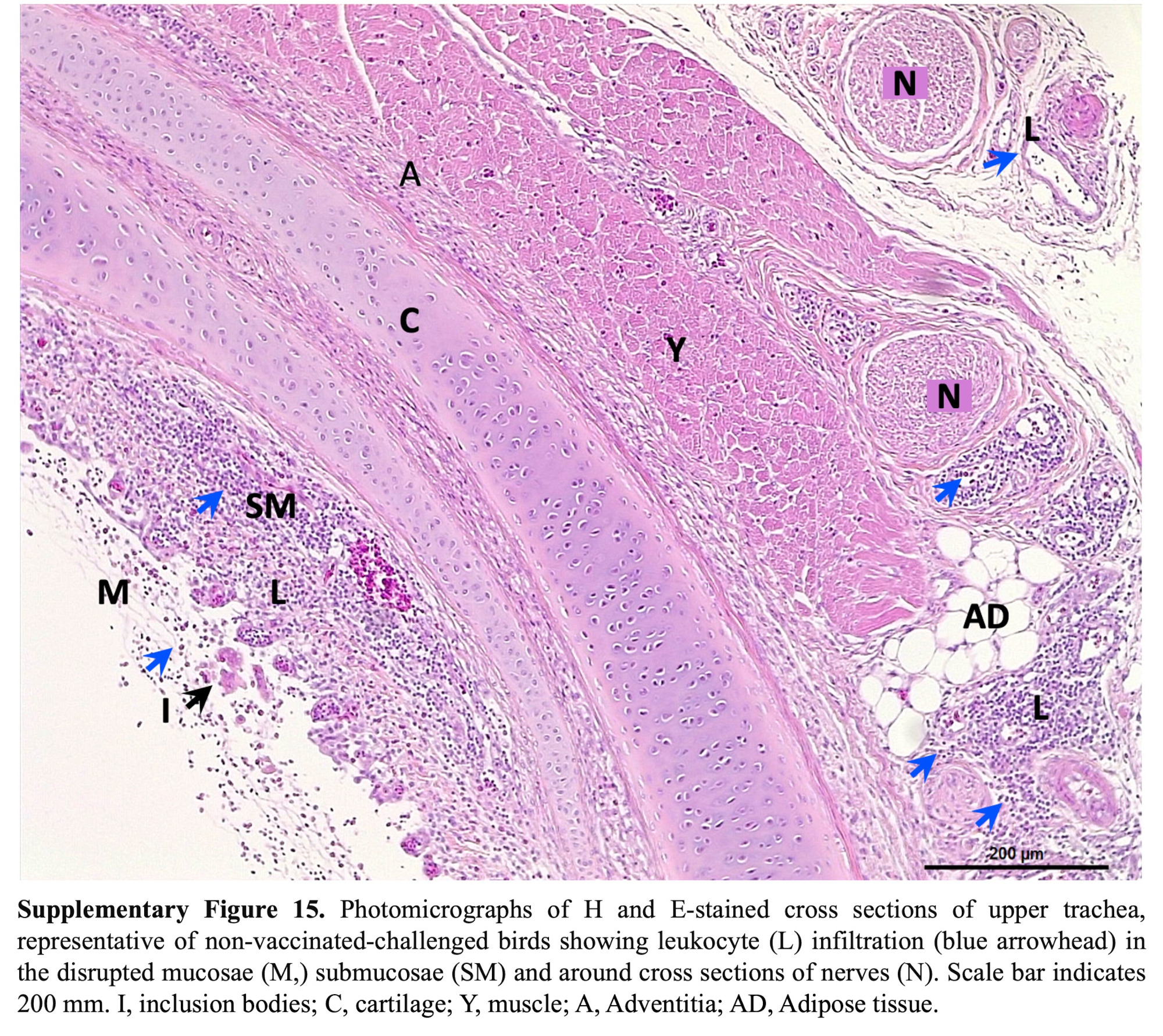

Supplement: Supplementary file 15 [file Image15.jpeg]
