# Supplementary material for: Clinical assessment and transcriptome analysis of host immune responses in a vaccination-challenge study using a glycoprotein G deletion mutant vaccine strain of infectious laryngotracheitis virus
Source: Front Immunol. 2025 Jan 24;15:1458218. doi: 10.3389/fimmu.2024.1458218 (PMC11802539; doi:10.3389/fimmu.2024.1458218)
Supplement: Supplementary file 16 [file Table1.docx]

**Supplementary Table 1.** Clinical signs scoring system used in this study.

| **Score** | **Description** |
| --- | --- |
| **Demeanour** | |
| 0 | Normal |
| 1 | Depressed demeanour |
| 2 | Severely depressed demeanour |
| **Dyspnoea** | |
| 0 | Normal breathing |
| 1 | Mild dyspnoea–beak remains closed |
| 2 | Moderate dyspnoea–open-beak breathing |
| 3 | Marked dyspnoea – gasping |
| 4 | Severe gasping. Birds dying during the trial scored as 4. |
| **Conjunctivitis** | |
| 0 | Conjunctival mucosa normal |
| 1 | Partial eye closure – mild conjunctivitis. |
| 2 | Complete eye closure – marked conjunctivitis |
