# Supplementary material for: Clinical assessment and transcriptome analysis of host immune responses in a vaccination-challenge study using a glycoprotein G deletion mutant vaccine strain of infectious laryngotracheitis virus
Source: Front Immunol. 2025 Jan 24;15:1458218. doi: 10.3389/fimmu.2024.1458218 (PMC11802539; doi:10.3389/fimmu.2024.1458218)
Supplement: Supplementary file 17 [file Table2.docx]

**Supplementary Table 2.** Tracheal gross pathology scoring system used in this study.

| **Score** | **Degree of tracheal gross pathology** |
| --- | --- |
| 0 | Absent. No pathology present |
| 1 | Mild amount of mucus present |
| 2 | Moderate amount of mucus present |
| 3 | Large amount of mucus present, some blood also present. Alternatively, diphtheritic material may be present but does not appear to block the trachea. |
| 4 | Large amount of mucus present, significant blood also present. Alternatively, a diphtheritic plug is present and blocks the trachea. |
