# Supplementary material for: Clinical assessment and transcriptome analysis of host immune responses in a vaccination-challenge study using a glycoprotein G deletion mutant vaccine strain of infectious laryngotracheitis virus
Source: Front Immunol. 2025 Jan 24;15:1458218. doi: 10.3389/fimmu.2024.1458218 (PMC11802539; doi:10.3389/fimmu.2024.1458218)
Supplement: Supplementary file 18 [file Table3.docx]

**Supplementary Table 3.** Tracheal histopathology scoring system used in this study.

| **Score** | **Histological findings** |
| --- | --- |
| 0 | Normal. Epithelium consists of thin pseudostratified columnar epithelium. Mucous glands normal |
| 1 | Minimal changes. Normal epithelium with mild to moderate infiltration of lymphocytes; heterophils rare. Mucous glands normal. No syncytia or cells with intra- nuclear inclusion bodies. |
| 2 | Mild changes. Mucosa thickened because of mild to moderate cell infiltration and/or epithelium essentially normal except for foci of syncytia with intranuclear inclusion bodies. Hyperemia, occasionally with cell cuffs. |
| 3 | Moderate changes. Mucosa thickened because of moderate to marked cell infiltration. Numerous syncytia with intranuclear inclusion bodies. Patches of affected epithelium often separating from, or, less commonly, sloughed from lamina propria. Mucosal surface well covered by normal or affected epithelium. Mucous glands reduced. Marked hyperemia, cuffs of mono- nuclear cells around vessels outside mucosa. |
| 4 | Sere changes. Mucosa thickened because of edema, proteinaceous fluid, cellular exudate, or adherent fibrinohemorrhagic to cellular pseudomembrane on the surface. Normal epithelium absent, mucosal sur- face covered by a thin layer of basal cells. Syncytia with inclusion bodies sometimes present. |
| 5 | Very severe changes. Same as 4, except mucosa has no residual epithelium and syncytia with inclusion bodies rarely found. |
