# Supplementary material for: Clinical assessment and transcriptome analysis of host immune responses in a vaccination-challenge study using a glycoprotein G deletion mutant vaccine strain of infectious laryngotracheitis virus
Source: Front Immunol. 2025 Jan 24;15:1458218. doi: 10.3389/fimmu.2024.1458218 (PMC11802539; doi:10.3389/fimmu.2024.1458218)
Supplement: Supplementary file 19 [file Table4.docx]

**Supplementary Table 4.** Mapping summary of the reads of the peripheral blood mononuclear cells collected from the uninfected and the vaccinated groups at 7 days post vaccination.

| Group | Sample ID | Total reads mapped to chicken genome  (%) |
| --- | --- | --- |
| Uninfected | L_1* | 41323508  (61.4) |
|  | L_2* | 42507851  (61.5) |
|  | L_3* | 43897367  (62.3) |
|  | L_4* | 39658002  (61.4) |
| Vaccinated | L_5**^¶^** | 46366295  (59.2) |
|  | L_6**^¶^** | 41638193  (60.5) |
|  | L_7**^¶^** | 44827466  (62.4) |
|  | L_8**^¶^** | 44036227  (61.2) |
|  | L_9 **^¶^** | 49546949  (64.4) |
|  | L_10**^¶^** | 37999107  (66.3) |
|  | L_11**^¶^** | 38142053  (59.9) |
|  | L_12**^¶^** | 43511358  (64.5) |

*Biological replicates of the Uninfected group

**^¶^**Biological replicates of the Vaccinated group
