# Supplementary material for: Clinical assessment and transcriptome analysis of host immune responses in a vaccination-challenge study using a glycoprotein G deletion mutant vaccine strain of infectious laryngotracheitis virus
Source: Front Immunol. 2025 Jan 24;15:1458218. doi: 10.3389/fimmu.2024.1458218 (PMC11802539; doi:10.3389/fimmu.2024.1458218)
Supplement: Supplementary file 20 [file Table5.docx]

**Supplementary Table 5.** Mapping summary of the reads of the tracheal scrapings collected from the uninfected, challenged-only and the vaccinated-challenged groups at 4- or 5-days post challenge.

| Group | Sample ID | Total reads mapped to chicken genome  (%) |
| --- | --- | --- |
| Uninfected | TS_1* | 108014316  (63.9) |
|  | TS_2* | 103007047  (63.4) |
|  | TS_3* | 79523930  (65.2) |
|  | TS_4* | 81323121  (65.6) |
|  | TS_5* | 94319973  (62.6) |
|  | TS_6* | 90661663  (66.7) |
| Challenged-only | TS_7**^†^** | 51607381  (41.1) |
|  | TS_8**^†^** | 76178870  (61.3) |
|  | TS_9**^†^** | 82633462  (48.5) |
|  | TS_10**^†^** | 109253432  (62.5) |
|  | TS_11**^†^** | 82690345  (61.4) |
|  | TS_12**^†^** | 92819102  (64.8) |
|  | TS_13**^†^** | 112492127  (55.5) |
|  | TS_14**^†^** | 95400169  (67.9) |
| Vaccinated-Challenged | TS_15 **^¶^** | 132158144  (67.2) |
|  | TS_16 **^¶^** | 92859192  (65.9) |
|  | TS_17 **^¶^** | 92323071  (64.4) |
|  | TS_18 **^¶^** | 79488577  (65.7) |
|  | TS_19 **^¶^** | 104111559  (65.3) |
|  | TS_20 **^¶^** | 109391734  (62.9) |

*Biological replicates of the Uninfected group

^†^Biological replicates of the Challenged-only group

^¶^Biological replicates of the Vaccinated-challenged group
