# Supplementary material for: Clinical assessment and transcriptome analysis of host immune responses in a vaccination-challenge study using a glycoprotein G deletion mutant vaccine strain of infectious laryngotracheitis virus
Source: Front Immunol. 2025 Jan 24;15:1458218. doi: 10.3389/fimmu.2024.1458218 (PMC11802539; doi:10.3389/fimmu.2024.1458218)
Supplement: Supplementary file 23 [file Table8.docx]

**Supplementary Table 8.** Genes with immune-related functions upregulated in the non-vaccinated-challenged group compared to the vaccinated-challenged or uninfected groups.

| **Gene name** | **Non-vaccinated-challenged *vs.*** | | | |
| --- | --- | --- | --- | --- |
|  | **Vaccinated-challenged** | | **Uninfected** | |
|  | **Padj**  **value** | **Log_2_**  **FC** | **Padj**  **value** | **Log_2_**  **FC** |
| ***Immunoglobulin- related*** | | |  |  |
| Immunoglobulin superfamily member 1 | 1.14E-30 | 5.98 | 3.92E-34 | 6.32 |
| Immunoglobulin-like receptor CHIR-AB1-like | 9.22E-24 | 5.68 | 9.07E-23 | 5.56 |
| Osteoclast-associated immunoglobulin-like receptor | 8.52E-13 | 5.08 | 1.50E-12 | 5.03 |
| Osteoclast-associated immunoglobulin-like receptor-like | 4.16E-09 | 4.60 | 1.53E-08 | 4.43 |
| Immunoglobulin-like receptor CHIR-B3 | 2.61E-12 | 4.01 | 9.87E-17 | 4.71 |
| Immunoglobulin superfamily member 1-like | 5.98E-09 | 4.01 | 1.03E-10 | 4.43 |
| Immunoglobulin-like receptor CHIR-B5 (CHIR-B5), mRNA. | 1.57E-07 | 3.91 | 8.59E-09 | 4.27 |
| Leukocyte immunoglobulin-like receptor subfamily A member 2 | 3.29E-08 | 3.87 | 5.70E-10 | 4.31 |
| Immunoglobulin superfamily member 6 | 3.10E-10 | 3.65 | 1.10E-15 | 4.56 |
| Gallus gallus immunoglobulin-like receptor CHIR-B6 | 2.13E-07 | 3.65 | 2.91E-08 | 3.91 |
| Osteoclast-associated immunoglobulin-like receptor (ENSGALG00000034411) | 6.42E-06 | 3.62 | 6.64E-07 | 3.97 |
| Leukocyte immunoglobulin-like receptor subfamily A member 2-like | 2.41E-06 | 3.34 | 9.40E-07 | 3.47 |
| V-set and immunoglobulin domain containing 4 | 1.93E-10 | 3.23 | 2.45E-12 | 3.52 |
| Gallus gallus immunoglobulin-like receptor CHIR-AB1 | 8.50E-05 | 3.23 | 3.50E-06 | 3.76 |
| Osteoclast-associated immunoglobulin-like receptor (ENSGALG00000045090) | 4.33E-05 | 3.23 | 3.12E-06 | 3.48 |
| Immunoglobulin-like receptor CHIR-B6 | 1.24E-04 | 3.12 | 4.18E-06 | 3.71 |
| Osteoclast-associated immunoglobulin-like receptor (ENSGALG00000046502) | 9.32E-04 | 2.47 | 2.35E-04 | 2.92 |
| Killer cell immunoglobulin-like receptor 3DL1 | 3.15E-03 | 2.44 | N | N |
| Gallus gallus immunoglobulin-like receptor CHIR-B2 | 2.55E-03 | 2.34 | 5.30E-03 | 2.19 |
| Leukocyte immunoglobulin-like receptor subfamily B member 4 | 8.49E-03 | 2.28 | 2.82E-03 | 2.57 |
| Osteoclast-associated immunoglobulin-like receptor (ENSGALG00000054710) | 6.99E-03 | 2.28 | 4.27E-03 | 2.43 |
| Immunoglobulin-like receptor CHIR-A2 | 9.61E-03 | 2.20 | N | N |
| Immunoglobulin-like and fibronectin type III domain containing 1 | 2.64E-03 | 1.96 | N | N |
| T-cell immunoglobulin and mucin domain containing 4 | 1.22E-03 | 1.39 | 3.60E-06 | 1.94 |
| Immunoglobulin-like receptor | 5.60E-03 | 1.21 | 8.57E-03 | 1.16 |
| polymeric immunoglobulin receptor-like | N | N | 4.96E-04 | 1.82 |
| ***Chemokines, Cytokines and receptors*** | | |  |  |
| interleukin 8-like 1 | 1.18E-24 | 7.04 | 2.29E-12 | 4.93 |
| interleukin 1, beta | 1.22E-21 | 6.40 | 3.34E-18 | 5.86 |
| interleukin 1 receptor type 2 | 2.56E-21 | 6.11 | 1.38E-19 | 5.85 |
| interleukin 5 receptor subunit alpha | 6.08E-14 | 5.74 | 1.91E-13 | 5.62 |
| interleukin 6 | 1.10E-16 | 5.72 | 6.30E-15 | 5.39 |
| interleukin-12 subunit beta-like | 3.04E-14 | 5.40 | 1.60E-12 | 5.02 |
| interleukin 4 induced 1 | 1.92E-12 | 5.36 | 1.10E-14 | 5.82 |
| interleukin 13 receptor subunit alpha 2 | 1.09E-19 | 5.12 | 3.95E-15 | 4.47 |
| interleukin 8-like 2 | 9.19E-12 | 4.95 | 6.25E-05 | 3.05 |
| interleukin 18 receptor accessory protein | 7.49E-15 | 4.69 | 2.67E-20 | 5.55 |
| interleukin 18 receptor 1 | N | N | 2.56E-03 | 1.25 |
| interleukin 22 | 2.70E-16 | 4.63 | 4.42E-16 | 4.60 |
| interleukin 10 receptor subunit alpha | 1.73E-11 | 4.01 | 3.44E-09 | 3.56 |
| interleukin 16 | 3.53E-11 | 3.91 | 1.88E-13 | 4.30 |
| interleukin 12B | 2.63E-07 | 3.64 | 1.65E-05 | 3.09 |
| interleukin 2 receptor subunit gamma | 5.65E-10 | 3.03 | 1.22E-13 | 3.57 |
| interleukin 12A | 2.73E-04 | 3.01 | 5.38E-03 | 2.34 |
| Interleukin 12 receptor subunit beta 2 | N | N | 4.47E-04 | 1.86 |
| interleukin 17F | 3.53E-05 | 2.87 | 2.62E-04 | 2.56 |
| interleukin 12 receptor subunit beta 1 | 5.42E-06 | 2.78 | 4.39E-08 | 3.30 |
| interleukin 10 | 6.06E-04 | 2.78 | N | N |
| interleukin 9 receptor | 7.81E-06 | 2.60 | 3.57E-08 | 3.14 |
| interleukin 4 | 2.46E-03 | 2.60 | 2.83E-03 | 2.56 |
| interleukin 17A | 2.42E-03 | 2.52 | N | N |
| interleukin 9 | 5.36E-03 | 2.41 | 2.08E-03 | 2.65 |
| interleukin 13 | 8.36E-03 | 2.25 | N | N |
| interleukin 3 | 9.67E-03 | 2.25 | N | N |
| interleukin 2 receptor subunit alpha | 4.85E-05 | 2.12 | 1.83E-04 | 1.9 |
| interleukin 2 receptor subunit beta | N | N | 8.50E-04 | 1.47 |
| interleukin 11 | 4.27E-04 | 2.11 | N | N |
| Interleukin 21 receptor | 6.41E-03 | 1.60 | 3.50E-07 | 2.85 |
| Interleukin 6 receptor | 1.27E-04 | 1.52 | 4.00E-03 | 1.17 |
| Interleukin 17 receptor A | 1.17E-04 | 1.43 | 2.39E-03 | 1.16 |
| Interleukin 22 receptor subunit alpha 2 | 3.16E-04 | 1.43 | N | N |
| Interleukin 20 receptor subunit alpha | 3.39E-03 | 1.41 | N | N |
| Interleukin 15 | 7.73E-03 | 1.39 | 7.66E-03 | 1.40 |
| Interleukin 7 receptor | 3.62E-03 | 1.37 | 8.62E-06 | 2.03 |
| Interleukin 7 | 1.73E-03 | 1.17 | 1.46E-04 | 1.41 |
| Interleukin 1 receptor accessory protein | 7.24E-04 | 1.11 | 1.11E-04 | 1.26 |
| C-X-C motif chemokine receptor 1 | 1.21E-31 | 6.63 | 3.92E-30 | 6.46 |
| C-C motif chemokine ligand 17 | 5.22E-33 | 6.38 | 1.15E-35 | 6.65 |
| Chemokine (C-C motif) ligand 5 | 4.66E-19 | 6.00 | 2.26E-15 | 5.36 |
| Chemokine ah221 | 4.61E-18 | 5.74 | 2.52E-16 | 5.44 |
| Chemokine-like ligand 4 | 1.81E-09 | 4.86 | 1.77E-09 | 4.86 |
| C-C motif chemokine ligand 26 | 1.32E-17 | 4.82 | 7.70E-21 | 5.26 |
| C-C motif chemokine receptor 2 | 2.64E-16 | 4.13 | 5.15E-24 | 5.05 |
| C-X-C motif chemokine ligand 13-like 2 | 2.15E-08 | 3.66 | 3.50E-07 | 3.35 |
| C-X-C motif chemokine ligand 13 | 6.69E-06 | 3.25 | 2.01E-09 | 4.20 |
| Chemokine (C-X-C motif) ligand 1-like | 2.08E-06 | 3.06 | N | N |
| C-X3-C motif chemokine receptor 1 | N | N | 2.08E-05 | 2.21 |
| Chemerin chemokine-like receptor 1 | 1.46E-07 | 2.88 | 1.87E-07 | 2.85 |
| C-X-C motif chemokine receptor 4 | 5.45E-08 | 2.37 | 1.11E-06 | 2.14 |
| C-C motif chemokine receptor 5 | 7.10E-06 | 2.27 | 1.92E-11 | 3.29 |
| C-C motif chemokine ligand 19 | 7.42E-08 | 2.26 | 9.85E-18 | 3.47 |
| C-X-C motif chemokine receptor 5 | 2.27E-03 | 1.86 | 1.71E-05 | 2.57 |
| C-C motif chemokine ligand 4 | 7.71E-03 | 1.70 | N | N |
| C-C motif chemokine receptor 8 | 1.71E-03 | 1.69 | 1.60E-05 | 2.29 |
| C-C motif chemokine ligand 1 | N | N | 3.57E-03 | 1.85 |
| C-C motif chemokine receptor 4 | 5.39E-04 | 1.67 | 3.73E-08 | 2.57 |
| leukocyte cell derived chemotaxin 2 | 6.20E-04 | 1.97 | 2.57E-04 | 2.09 |
| X-C motif chemokine ligand 1 | N | N | 2.07E-04 | 1.90 |
| C-C motif chemokine receptor 7 | 4.93E-03 | 1.49 | 1.30E-03 | 1.70 |
| TAFA chemokine like family member 3 | 3.95E-03 | 1.34 | N | N |
| Cytokine receptor common subunit beta-like | 1.99E-29 | 5.98 | 2.28E-33 | 6.39 |
| Suppressor of cytokine signaling 1 | 4.66E-27 | 5.48 | 1.84E-15 | 5.0 |
| Cytokine inducible SH2 containing protein | 9.72E-06 | 2.19 | N | N |
| Suppressor of cytokine signalling 1-like protein | 9.21E-06 | 2.06 | 3.16E-06 | 2.15 |
| Suppressor of cytokine signaling 3 | 4.88E-03 | 1.86 | N | N |
| Cytokine receptor-like factor 2 | 3.83E-03 | 1.62 | 5.07E-05 | 2.23 |
| Cytokine receptor like factor 3 | 2.82E-05 | 1.17 | 2.98E-08 | 1.50 |
| ***TLR*** | | |  |  |
| Toll-like receptor 15 | 4.89E-20 | 6.27 | 4.29E-20 | 6.27 |
| Toll like receptor 4 | 3.72E-21 | 5.60 | 1.28E-23 | 5.94 |
| Toll like receptor 7 | 3.44E-07 | 3.02 | 3.42E-13 | 4.18 |
| Toll-like receptor 2 family member B | 2.61E-06 | 2.43 | 3.11E-09 | 3.03 |
| Toll-like receptor 1 family member B | 1.77E-06 | 1.93 | 1.58E-05 | 1.80 |
| Toll-like receptor 2 family member A | 6.45E-05 | 1.73 | 4.45E-06 | 1.96 |
| ***MHC*** | | |  |  |
| Major histocompatibility complex class II beta chain BLB1 | 2.31E-07 | 2.74 | 1.56E-09 | 3.15 |
| Major histocompatibility complex class II beta chain BLB2 | 6.48E-07 | 2.57 | 8.95E-10 | 3.08 |
| Major histocompatibility complex, class II, DM beta 2 | 4.49E-06 | 2.32 | 3.47E-10 | 3.07 |
| Major histocompatibility complex, class II, DM alpha | 1.20E-03 | 1.65 | 1.02E-08 | 2.78 |
| Major histocompatibility complex class I antigen BF2 | 2.08E-06 | 1.53 | 2.89E-06 | 1.50 |
| Major histocompatibility complex, class II, DM beta 1 | 2.88E-03 | 1.45 | 1.58E-07 | 2.46 |
| Class II major histocompatibility complex transactivator | 6.22E-04 | 1.33 | 1.84E-05 | 1.64 |
| MHC B-G antigen | 1.89E-05 | 2.30 | 1.09E-03 | 1.80 |
| MHC BF1 class I | 2.20E-06 | 1.72 | 7.40E-07 | 1.79 |
| MHC class I antigen YF5 | 9.14E-05 | 1.03 | 5.86E-05 | 1.06 |
| MHC-like class I Y | 5.72E-05 | 1.00 | 2.70E-05 | 1.04 |
| Major histocompatibility complex, class I, A6 | N | N | 1.65E-03 | 1.10 |
| ***Interferon- related*** | | |  |  |
| Interferon kappa-like 1 | 5.52E-12 | 5.08 | 1.68E-11 | 4.97 |
| Interferon gamma | 5.65E-10 | 4.51 | 1.49E-08 | 4.14 |
| Interferon induced protein with tetratricopeptide repeats 5 | 8.87E-24 | 4.22 | 8.83E-20 | 3.84 |
| Interferon alpha inducible protein 6 | 3.67E-19 | 3.18 | 2.93E-18 | 3.10 |
| Interferon regulatory factor 5 | 1.22E-11 | 3.13 | 2.52E-16 | 3.72 |
| Interferon induced with helicase C domain 1 | 6.39E-12 | 2.50 | 2.56E-09 | 2.19 |
| Interferon regulatory factor 9 | 1.68E-09 | 2.37 | 1.82E-08 | 2.23 |
| Interferon omega 1 | 7.79E-03 | 2.30 | N | N |
| Interferon-induced transmembrane protein 3-like | 7.39E-04 | 2.10 | N | N |
| Interferon-induced transmembrane protein 1-like | 2.37E-16 | 1.94 | 3.50E-16 | 1.92 |
| Interferon regulatory factor 4 | 3.35E-04 | 1.87 | 3.17E-03 | 1.55 |
| Interferon alpha and beta receptor subunit 1 | 9.72E-05 | 1.77 | 2.41E-04 | 1.67 |
| Interferon regulatory factor 1 | 5.23E-04 | 1.63 | N | N |
| Interferon induced transmembrane protein 5 | 1.65E-03 | 1.46 | N | N |
| Interferon regulatory factor 7 | 4.46E-04 | 1.37 | N | N |
| Interferon, alpha-inducible protein 27-like 2 | 5.13E-05 | 1.35 | 1.54E-03 | 1.08 |
| GTPase, very large interferon inducible pseudogene 1 | 7.37E-16 | 4.54 | 7.27E-21 | 5.23 |
| Myxovirus (influenza virus) resistance 1, Interferon-inducible protein p78 (mouse) | 7.80E-11 | 1.81 | 3.11E-08 | 1.56 |
| Interferon regulatory factor 8 | N | N | 6.90E-06 | 1.08 |
| interferon-induced protein 35 | N | N | 2.39E-08 | 1.05 |
| ***Nuclear factor (NF)-related*** | | |  |  |
| Nuclear factor, erythroid 2 | 2.16E-04 | 1.80 | 1.90E-03 | 1.53 |
| Nuclear factor, interleukin 3 regulated | 1.08E-06 | 1.37 | N | N |
| Nuclear factor kappa B subunit 2 | 5.68E-03 | 1.18 | N | N |
| ***Tumor necrosis factor (TNF)- related*** | | |  |  |
| TNFAIP3 interacting protein 2 | 1.27E-08 | 4.02 | 6.39E-05 | 2.92 |
| TNF receptor superfamily member 1B | 4.99E-11 | 3.31 | 4.71E-11 | 3.31 |
| Fas ligand (TNF superfamily, member 6) | 4.97E-08 | 2.63 | 8.97E-04 | 1.66 |
| TNF receptor superfamily member 4 | 7.39E-07 | 2.48 | 5.61E-05 | 2.05 |
| TNF receptor superfamily member 25 | 1.13E-05 | 2.16 | 3.19E-03 | 1.50 |
| TNF receptor superfamily member 18 | 1.16E-04 | 1.74 | 1.10E-08 | 2.48 |
| TNF receptor associated factor 1 | 1.22E-04 | 1.59 | 1.32E-04 | 1.58 |
| TNFAIP3 interacting protein 3 | 1.92E-03 | 1.50 | N | N |
| Lipopolysaccharide induced TNF factor | 1.18E-07 | 1.23 | 9.90E-09 | 1.32 |
| Tumor necrosis factor superfamily member 11 | 2.96E-10 | 3.56 | 2.01E-06 | 2.75 |
| Tumor necrosis factor superfamily member 13b | 2.36E-07 | 2.82 | 5.51E-09 | 3.14 |
| Tumor necrosis factor superfamily member 8 | 1.47E-07 | 2.55 | 1.26E-09 | 2.90 |
| ***Complement*** | | |  |  |
| Complement C5a receptor 1 | 2.82E-16 | 5.61 | 1.28E-14 | 5.30 |
| Complement C3a receptor 1 | 1.74E-11 | 3.35 | 2.52E-16 | 4.02 |
| Complement C1q A chain | 3.51E-12 | 3.30 | 2.16E-16 | 3.85 |
| Complement C1q C chain | 4.64E-12 | 3.27 | 9.16E-16 | 3.75 |
| Fanconi anemia complementation group A | 1.87E-09 | 2.40 | 2.73E-12 | 2.76 |
| Complement C1q B chain | 2.77E-07 | 2.32 | 4.01E-12 | 3.05 |
| Complement component 3 | 9.55E-06 | 1.61 | 1.93E-05 | 1.56 |
| Complement 4 | 1.81E-04 | 1.17 | 8.05E-06 | 1.37 |
| Fanconi anemia complementation group C | 5.93E-04 | 1.12 | 5.29E-04 | 1.13 |
| ***Cluster of differentiation (CD)*** | | |  |  |
| CD300 molecule like family member g | 2.05E-20 | 4.84 | 1.74E-25 | 5.43 |
| CD1c molecule | 3.21E-17 | 3.98 | 8.48E-21 | 4.40 |
| CD80 molecule | 1.63E-12 | 3.95 | 2.72E-11 | 3.73 |
| CD86 molecule | 6.31E-12 | 3.85 | 5.14E-14 | 4.24 |
| CD72 antigen | 9.41E-08 | 3.82 | 5.73E-10 | 4.37 |
| CD48 molecule | 1.20E-13 | 3.80 | 1.55E-20 | 4.68 |
| CD180 molecule | 5.03E-12 | 3.56 | 3.50E-14 | 3.90 |
| CD1b molecule | 3.15E-12 | 3.41 | 1.44E-18 | 4.23 |
| CD200 receptor 1 | 1.46E-09 | 3.22 | 5.15E-08 | 2.92 |
| CD40 molecule | 1.73E-06 | 2.24 | 1.04E-05 | 2.08 |
| CD40 ligand | 1.48E-04 | 2.01 | 3.36E-04 | 1.91 |
| CD247 molecule | 6.50E-06 | 1.92 | 2.23E-05 | 1.80 |
| CD38 molecule | 1.56E-04 | 1.50 | 1.67E-08 | 2.15 |
| CD74 molecule | 1.91E-03 | 1.41 | 1.39E-09 | 2.60 |
| CD63 molecule | 8.13E-05 | 1.06 | N | N |
| CD79b molecule | N | N | 1.35E-05 | 2.55 |
| CD5 molecule | N | N | 1.93E-05 | 2.53 |
| CD8b molecule pseudogene | N | N | 1.75E-04 | 2.09 |
| CD3d molecule | N | N | 1.08E-05 | 2.02 |
| CD4 molecule | N | N | 7.05E-06 | 2.04 |
| CD8a molecule | N | N | 9.70E-05 | 1.82 |
| CD7 molecule | N | N | 7.60E-04 | 1.62 |
| CD226 molecule | N | N | 5.44E-03 | 1.41 |
| CD81 molecule | N | N | 1.72E-06 | 1.04 |
| CD3e molecule | N | N | 1.21E-03 | 1.00 |

Padj value < 0.01and log_2_(1) FC (2-fold change) ≥1 was considered significant; N, not upregulated.
