# Supplementary material for: Clinical assessment and transcriptome analysis of host immune responses in a vaccination-challenge study using a glycoprotein G deletion mutant vaccine strain of infectious laryngotracheitis virus
Source: Front Immunol. 2025 Jan 24;15:1458218. doi: 10.3389/fimmu.2024.1458218 (PMC11802539; doi:10.3389/fimmu.2024.1458218)
Supplement: Supplementary file 24 [file Table9.docx]

**Supplementary Table 9.** Immune related genes downregulated in the non-vaccinated-challenged group compared to the vaccinated-challenged and challenged only group

| **Gene name** | **Non-vaccinated-challenged *vs.*** | | | |
| --- | --- | --- | --- | --- |
|  | **Vaccinated-challenged** | | **Uninfected** | |
|  | **P-adj**  **value** | **Log_2_**  **FC** | **P-adj**  **value** | **Log_2_**  **FC** |
| ***Immunoglobulin- related*** | | |  |  |
| V-set and immunoglobulin domain containing 1 | 9.40E-06 | -2.05 | 1.11E-03 | -1.54 |
| Immunoglobulin superfamily containing leucine rich repeat | 1.22E-04 | -1.34 | 4.43E-04 | -1.23 |
| Immunoglobulin superfamily member 11 | 8.99E-05 | -1.11 | N | N |
| immunoglobin superfamily member 21 | N | N | 5.11E-03 | -1.00 |
| ***Chemokines, Cytokines and receptors*** | | |  |  |
| Interleukin 17 receptor E like | 2.33E-06 | -2.94 | 8.90E-04 | -2.13 |
| Interleukin 1 receptor accessory protein like 2 (IL1RAP/IL1R3) | 6.97E-05 | -2.10 | 1.17E-03 | -1.74 |
| Interleukin 1 receptor type 1 | 1.21E-03 | -1.07 | 1.02E-03 | -1.08 |
| C-C motif chemokine ligand 20 | 1.31E-05 | -1.86 | 8.62E-10 | -2.52 |
| ***Complement-related*** | | |  |  |
| Complement component 1, q subcomponent-like 2 | 8.78E-07 | -2.41 | N | N |
| Complement factor H | 3.66E-04 | -1.80 | 4.09E-04 | -1.78 |
| Complement factor D | 1.73E-04 | -1.69 | N | N |
| Complement C4A | 3.30E-04 | -1.31 | N | N |
| C1q and tumor necrosis factor related protein 8 | 1.02E-06 | -3.00 | 2.53E-07 | -3.14 |
| C1q and tumor necrosis factor related protein 2 | 2.03E-04 | -2.08 | 2.06E-04 | -2.08 |
| C1q and TNF related 7 | 1.57E-05 | -1.95 | 3.04E-03 | -1.38 |
| C1q and tumor necrosis factor related protein 5 | 1.18E-03 | -1.30 | 8.22E-05 | -1.56 |
| Adiponectin, C1Q and collagen domain containing | 6.64E-03 | 1.02 | 7.65E-04 | -1.25 |
| ***Cluster of differentiation (CD)*** | | |  |  |
| CD34 molecule | 7.58E-03 | -1.164 | 3.24E-03 | -1.28 |
| CD109 molecule | 2.57E-03 | -1.127 | 1.03E-04 | -1.43 |
| CD24 molecule | 1.97E-04 | -1.034 | 2.20E-04 | -1.03 |

Padj value < 0.01 and log_2_ (1) FC (2-fold change) ≤ - 1 was considered significant; N, not downregulated.
